# Microheterogeneous Catalysis

**DOI:** 10.3390/molecules15074815

**Published:** 2010-07-09

**Authors:** Eva Bernal, María Marchena, Francisco Sánchez

**Affiliations:** Department of Physical Chemistry, University of Seville, C/Profesor García González, s/n, 41012, Seville, Spain; E-Mails: evabernal@us.es (E.B); marijose@us.es (M.M)

**Keywords:** microheterogeneous catalysis, micelle, polymer, cyclodextrin, photochemistry

## Abstract

The catalytic effect of micelles, polymers (such as DNA, polypeptides) and nanoparticles, saturable receptors (cyclodextrins and calixarenes) and more complex systems (mixing some of the above mentioned catalysts) have been reviewed. In these microheterogeneous systems the observed changes in the rate constants have been rationalized using the Pseudophase Model. This model produces equations that can be derived from the Brönsted equation, which is the basis for a more general formulation of catalytic effects, including electrocatalysis. When, in the catalyzed reaction one of the reactants is in the excited state, the applicability (at least formally) of the Pseudophase Model occurs only in two limiting situations: the lifetime of the fluorophore and the distributions of the quencher and the probe are the main properties that define the different situations.

## 1. Introduction

This review deals with catalysis in microheterogeneous systems, that is, in systems in which one (or several) phases are dispersed in another phase (the bulk phase), the dispersed phase being the catalyst. Micellar solutions and microemulsions are typical examples of the systems under consideration.

Generally speaking, the amount of bulk phase in microheterogeneous systems is much greater than the amount of the dispersed phase. This implies that, in order to have effective catalytic activity, the affinity of the dispersed phase by the reactant(s) must be considerably greater than that of the bulk phase, in such a way that a significant part of the reactant(s) is incorporated into the dispersed phase. This affinity results from a variety of interactions such as electrostatic, hydrogen bonding, hydrophobic, π-stacking, *etc.*

Therefore, if a significant part of the reactant is present in the dispersed phase (catalyst *C*) the possibility of two reaction paths appears. For the case in which the reaction is slow, compared to the kinetics of the distribution of the reactant(s) between the different phases, one can assume that this distribution is at equilibrium:

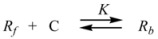
(1)
in such a way that two populations of reactants, free, *R_f_*, and bound to the catalyst, *R_b_*, are present in the system. These populations react at different rates, so that a catalytic effect appears.

Several causes can produce an increase in the reaction rate of the bound species. Thus, for bimolecular reactions, if the two reactants are preferentially bound to the dispersed phase, an increase in the local concentrations of these reactants will be observed. This concentration effect will produce an increase in the reaction rate [1]. However, this effect cannot be the cause of the differences of reactivity for the free and bound reactants in the case of unimolecular reactions. For this kind of process, the differences must be related to the properties of the local reaction media that, generally speaking, are quite different from the properties of the bulk phase. So, in the case of solutions containing charged micelles intense local electric fields appear. These fields affect the local properties and, thus, the reaction rate. For example, in the case of electron transfer reactions, the solvent reorganization energy depends on the dielectric characteristic of the surrounding medium [2], and these characteristics are modified by the electric field through solvent saturation effects [3]. On the other hand, the reaction free energy is dependent on the field, because the free energies of the reactant and product states also depend on the dielectric constant of the media. Moreover, the field can change the adiabaticity of the reaction through the polarization of the orbitals of the reactants involved in the electron transfer [4]. The dynamics of the solvent, and thus the pre-exponential term in the rate constant are also changed by the field [5,6]. Indeed, the diffusion coefficients of the intervening species, corresponding to the non homogeneous state (in the presence of the field), are quite different from those of the homogeneous state (without the field) [6]. Therefore, the equilibrium correlations, such as the direct correlation functions, in the presence of a field may also be rather different from those in the absence of the field [7]. Finally, it has been suggested that the fluctuation-dissipation theorem and other important theorems of statistical mechanics may no longer be valid in the presence of a strong field [8,9,10]. All the above mentioned effects can produce dramatic changes in the rate and characteristics of electron transfer reactions in microheterogeneous systems.

Thus, the electron transfer reaction rate within the binuclear pentammineruthenium (III) (μ-cyano)pentacyano-ruthenium (II) complex increases by a factor of about 100 in the presence of hexadecyltrimethylammonium chloride micelles [11]. On the other hand Choudhury *et al*. [12] claimed that the electron transfer reactions between some coumarin derivatives and amines change from the normal to the inverted regime [13] in micellar solutions.

Of course, electron transfer reactions are neither the only kind of reaction influenced by microheterogeneous catalysis nor the only one that can be influenced by the effects mentioned above. Thus, in the field of Inorganic Chemistry, ligand substitution reactions are also influenced [14,15], as are organic reactions [16] or solvolytic processes [17,18]. Moreover, the influence of microheterogeneous catalysis is not limited to the change of the reaction rate. In some cases a change of the products of a given reaction (which, of course implies a change in the reaction mechanism) has been observed [19].

The effects mentioned above operating in microheterogeneous catalysis indicate clearly the complex character of this type of process. Elucidation of the main factors influencing each particular example is a challenge, which makes the study important from a fundamental point of view.

On the other hand, microheterogeneous catalysis can offer, at least in some cases, advantages in relation to other types of catalysis. Thus, frequently this kind of process can be carried out under average conditions. It is also interesting that this type of reaction does not require, generally speaking, complicated manipulation systems. Moreover, there are many experimental techniques that can provide reliable information on the state of the reactant and the catalyst [17,18]. Finally, an important aspect of microheterogeneous catalysis is its versatility. For example, in systems constituted by surfactants, an external stimulus (light, a variation of pH, *etc*.) can change the structure of the catalyst drastically: fatty acid soaps aggregate into micelles in a high pH buffer while a simple drop in pH leads to a transformation into bilayer vesicles [20]. In fact, by using this property, the formation of lipid vesicles from micelles and their controlled continuous growth has been achieved with fatty acids [21]. Another example of the versatility of this kind of system comes from the field of protein extraction using microemulsions. In this case, electrostatic interactions compete with biospecific interactions and can disturb the selectivity of the extractive process. However electrostatic interactions can be reduced by doping the interface with non-ionic surfactants [20]. Another way of modulating the balance of electrostatic and other kinds of interactions is to add electrolytes to the systems [22].

The previous paragraphs shows the interest in microheterogeneous catalysis from a basic as well as from an applied point of view. In the following sections of this review this topic will be considered according to the following organization. In Section 2 a common formulation describing homogeneous, heterogeneous and electrocatalysis is presented. Section 3 describes the effects of microheterogeneous catalysts on ground state reactions. These effects, on photochemical reactions, are considered in Section 4. Section 5 gives the general conclusions of this review.

## 2. A Common Formulation for Homogeneous, Heterogeneous Catalysis and Electrocatalysis

Variations of the rate of a given reaction in the presence of a microcatalyst or a salt, when the concentration of the catalyst or the salt are changed, can be described by equations that are formally identical [23].These variations, in fact, are described by the equation of the Pseudophase Model [24] and the Olson-Simonson equation [25], which are isomorphous. 

In both cases, it is supposed that the reactant(s) are present in two states (see Equation 1), which are at equilibrium even if they participate in a chemical reaction, that is, the forward and the reverse processes in Equation 1 are much more rapid than the reactions in which* R_f_* and *R_b _*participates. Under these circumstances, if *C* (the catalyst) is in excess, the concentrations of free and bound reactants, in the system, are given by [26]:

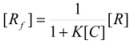
(2a)

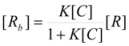
(2b)


If these two states react at different rates, the observed rate constant, *k_obs_*, is given by:

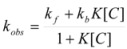
(3)
where *k_f_* and *k_b_* are the rate constants corresponding to the reactions of the free and bound states of *R*, respectively.

Equation 3 is the equation of the Pseudophase Model when [*C*] is the concentration of the dispersed pseudophase (the catalyst), or the Olson-Simonson equation if [*C*] represents the concentration of the salt influencing the reaction. Of course, this equation also describes the observed behavior of the homogeneous catalysis, provided that the catalyst is in excess over the reactant. 

A few years after the publication of Olson and Simonson’s paper, Scatchard [27] showed that Equation 3 can be derived from the Brönsted equation [28]. This equation is:

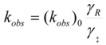
(4a)


or:

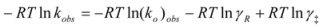
(4b)


or:

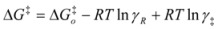
(4c)


In this equation (*k_o_*)*_obs _*is the rate constant in an (arbitrary) reference state and γ_R_ and γ_‡_ are the activity coefficients of the reactant and transition state, corresponding to the selected reference state. 

**Scheme 1:**
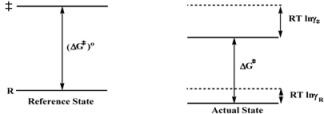
Schematic Representation of the changes in the activation free energy of a reaction when the system goes from a reference state to the actual state.

The Brönsted equation is, in some sense an identity because *RT*lnγ_i_ (*i* = *R*, ‡) is the change in free energy of *i* when going from the reference state to the actual state, in which the rate constant is *k_obs_*. According to this, Equation 4c establishes that the difference between the free energy of the transition state and the free energy of the reactant, in the actual state, is the same as this difference in the reference state plus the change in the free energy of the transition state minus the free energy of the reactant when going from the reference to the actual state*.* This statement (see Scheme 1) describes an obvious fact.

Given the identity characteristic of the Brönsted equation, it is clear that it will describe changes in reactivity when going from the reference to the actual state, provided that the Transition State Theory holds. In the case of catalytic reactions an additional condition is that, as mentioned previously, the process in Equation 1 remains at equilibrium in the reacting systems.

Now the relationship between Equation 3 and the Brönsted equation will be established [29]. For this purpose consider a solution of reactant *R*, and some species, *C*, able to bind to *R* (Equation 1). *C* can be a catalyst or a counterion. The contribution of *R* to the free energy of the solution is given by:

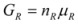
(5a)


or:

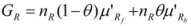
(5b)



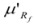
 being the chemical potential of free *R* and 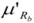 the chemical potential of *R* bound to the catalyst (see Equation 1). *θ* represents the association degree of *R* to *C*.

In Equation 5a, *n_R_* is the total number of moles of *R* in the solution and *μ_R_* its chemical potential. Equation 5b has been written taking into account the possibility of binding *R* to *C*.

It is clear from Equations 5a and 5b that:

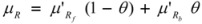
(6)
but free and bound *R* are at equilibrium. So their chemical potentials are the same, that is:

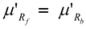
(7)


This, taking into account Equation 6, implies that:

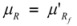
(8)


That is:

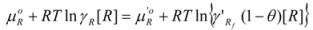
(9)


On the other hand, it is clear that 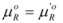 and thus:

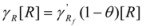
(10)

From this equation it follows that:

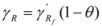
(11)

Consequently, the experimental activity coefficient γ_R_ would be the product of the activity coefficient of free reactants, γ_Rf _times the degree of dissociation. If one takes as the reference state the free *R* (and thus γ'_Rf_ = 1), Equation 11 results in:

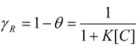
(12)


Now consider that the two forms of *R* (free and bound) can react to give a product. According to the Brönsted equation, the rate constant would be:

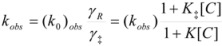
(13)


But (*k_0_*)*_obs_* is just *k_f_*, because the reference state is the free ions, so:

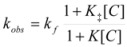
(14)


On the other hand, 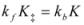 (see reference 29), consequently Equations 3 and 14 are the same. In the case of homogeneous or microheterogeneous catalysis, the rate of reaction according to previous equations would be given by:

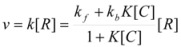
(15)
where [R] represents the total concentration of the reactant in the system.

If the non-catalyzed reaction path is not significant, one can write:


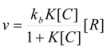
(16)

The maximum value of *ν* corresponds to the case in which the reactant is completely bound to the reactant, in such a way that [*R*] = [*R_b_*]:

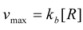
(17)


Writing now 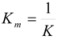, Equation 16 results in:

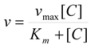
(18)


This equation is similar to the Michaelis-Menten equation, and, as was demonstrated, can be obtained from the Brönsted equation. However, Equation 18 is applicable when *C* is in excess over *R*, because this is the condition to apply previous equations and, in particular, Equation 12.

The case of heterogeneous catalysis will be treated now. In particular, the case in which the catalyst is a solid and the reactant is a gas will be considered. In order to use the Brönsted equation, the adsorption/desorption processes will be assumed rapid compared to the reaction step, that is, the equilibrium condition holds.

Under these circumstances, taking as the reference state the free gas, one can write:

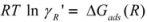
(19a)

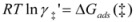
(19b)


If *K_R_* and 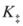 are the equilibrium constants corresponding to the adsorption of the reactant and transition state respectively, it is clear that:

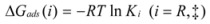
(20)
that is:

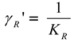
(21a)

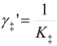
(21b)


From the Brönsted equation, the rate constant corresponding to the reaction of the adsorbed reactant 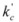 is given by:

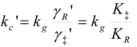
(22)
where *k_g_* is the rate constant for the non-adsorbed reactant, that is, the reactant in the reference state, the gas phase. From Equation 22 it follows:

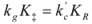
(23)


Consider now a system at constant volume and temperature, in such a way that concentration and number of moles of a gas contained in this volume are proportional. This volume contains the gas (the reactant *R*) that can be adsorbed on a surface contained in the volume (the catalyst) which has *N* adsorption sites. Under these circumstances, the following process happens:

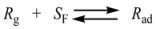
(24)
where Ѕ_F_ represents a free site on the catalyst. Thus, it can be written:

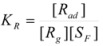
(25)
with the concentrations referred to the (fixed) volume of the system.

If the gas experiences a reaction (slow enough to maintain the adsorption equilibrium, Equation 24) it is possible to write:

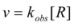
(26a)


or :

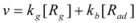
(26b)


Of course:

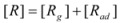
(27)


From the previous equations it follows that:

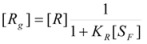
(28a)

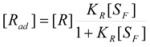
(28b)
and thus:

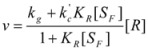
(29)


So that:

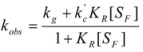
(30)


Equation 30 can be obtained from the Brönsted equation, taking into account that the activity coefficients of the reactant, *R*, and the transition state, ‡, in the system (not in the adsorbed state, as given by Equations 21) are:

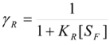
(31a)

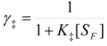
(31b)


Thus:

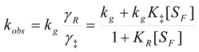
(32)


But this equation, taking into account Equation 23, is the same as Equation 30. Now consider again Equation 29. Assuming that the rate of the non-catalyzed reaction is negligibly small one can write:

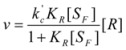
(33)
or (see Equation 28b):

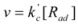
(34)


Of course, concentration of the adsorbed reactant is the same as concentration of occupied sites, if each reactant molecule adsorbs while occupying only one site. Thus:

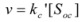
(35)


Taking into account that:

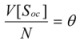
(36)


*θ* being the fraction of the covered surface, it follows:

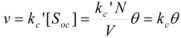
(37a)

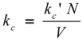
(37b)


If the adsorption of the gas is of the Langmuir type, *θ* is given by [30,31]:

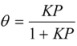
(38)
and thus:

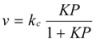
(39)
which is the familiar equation for the rate of a catalyzed reaction when the reactant gas is adsorbed on the catalyst with a Langmuir type adsorption isotherm.

Finally, the case of electrocatalysis will be considered. This is the term frequently used by electrochemists to describe the effect of the electrode potential on the kinetics of electrode reactions [32]. The rate of these reactions is measured as the current density, that is, the current per unit area of the electrode.

In the following paragraphs, the fundamental equation of the electrocatalysis, that is, the Butler-Volmer equation will be derived from the Brönsted equation. For this purpose, consider an electrode process in which an electron acceptor *A* is reduced at the electrode:

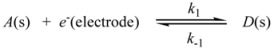
(40)


*D* represents the reduced *A*, that is, an electron donor. For simplicity, the charges on *A* and *D* are not written. Of course, they have a unit difference in their charges. 

The net rate of reaction expressed as the current density is:

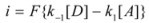
(41)
where *F* is the Faraday constant and the anodic current is considered to be positive.

Taking as the reference states for *D* and *A* the states of these species in the actual solution when the electrode potential difference is zero, that is, when the potential of the metal (electrode) and the solution are the same, the chemical potentials of *A* and *D* when the solution changes its potential (with respect to the metal) are given by:

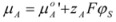
(42a)

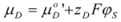
(42b)


Assuming that the transition state bears a charge 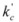, its chemical potential will be:

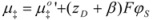
(43)


It is clear that the activity coefficients of *D*, *A* and transition state, with respect to the selected reference state, are given by:

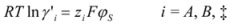
(44)


According to this, the variation of *φs* will imply a change in the activation free energy, and thus, a change in the rate constant. According to the Brönsted equation, the changes are given by:

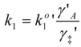
(45a)

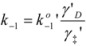
(45b)



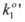
 y 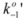 being the rate constants at the reference state, that is when *φ_S_* = *φ_M_*.

From Equations 42 to 45 it follows that:

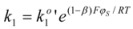
(46a)

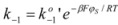
(46b)


In this way Equation 41 becomes:

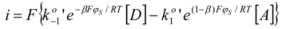
(47)


Eliminating the condition of constant φ_M_, this equation would be written, in terms of ∆*φ*′ = *φ_s_* −*φ_M_* as:

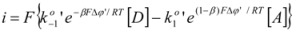
(48)
or, as is customary in the electrochemical context:

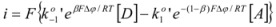
(49)
with ∆*φ* = −∆*φ*′ = *φ_M_* −*φ_s_*.

When there is no current at the electrode, ∆*φ* reaches its equilibrium value, ∆*φ_e_*. In this case:

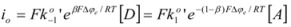
(50)


In terms of *i_0_* and the overpotential, *η* = ∆*φ* − ∆*φ_e_*, Equation 49 reads:

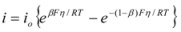
(51)


This is the Butler-Volmer equation [33,34], the fundamental equation of electrocatalysis. On the other hand, using Equation 50 it is possible to express ∆*φ_e_*, that is the equilibrium electrode potential, as given by the Nernst equation, in terms of 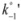 and 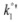:

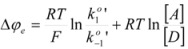
(52)


Moreover, as established above, 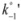 and 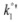 correspond to a reference state (the actual solution when ∆*φ* = 0) different from the customary one. These rate constants can be written as:

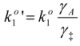
(53a)

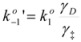
(53b)


In terms of the rate constant, 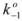 and 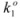, and the activity coefficients, referred to the customary reference state (of infinite dilution). In this way:

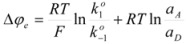
(54)
or:

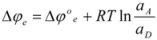
(55)


In Equation 55, 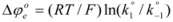 and *α_A_* and *α_D_* are the activities of the acceptor and donor, respectively.

Of course, in the application of the Nernst equation, ∆*φ_e_* is measured with respect to the potential of a reference electrode, ∆*φ_ref_*, in such a way that it produces:

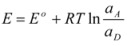
(56)


*E* being ∆*φ* − ∆*φ_ref_* and *E*° being 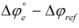.

Thus it has been demonstrated that both the Butler-Volmer equation as well as the Nernst equation can be deduced from the Brönsted equation. In fact, the Nernst equation offers the possibility of checking the treatment developed in this Section and in particular Equation 12 in which this treatment is based. Thus, Equation 56 can be written as:

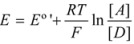
(57)
where *E*°′ is the so-called standard formal redox potential and is given by:

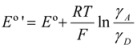
(58)


This equation, using Equation 12, becomes:

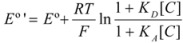
(59)


Equation 59 has been checked recently by one of us through the measurements of the standard formal redox potentials of the redox couple [Fe(CN)_5_(4-CNpy)]^2−^^/3^^−^ (4-CNpy = 4-cyanopyridine) in the presence of micelles of hexadecyltrimethylammonium chloride (CTAC) [35]. From these results it is clear that Equation 59 describes well the changes in the standard formal redox potential when the concentration of micelles is varied. It is interesting to note that Equation 59 permits us to calculate the binding constant of reactants to a microcatalyst (receptor) through measurements of standard formal redox potentials, as Almgren *et al*. pointed out [36]. On the other hand, changes in the half wave potentials of redox couples in micellar solutions are in agreement with previous equations [37], as are the changes in the equilibrium constants for the reactions of ferrocene, Fe(Cp)_2_, with a series of cobalt complexes in micellar solutions, when the concentration of micelles is changed [38].

Thus, it is possible to conclude that the previous treatment has been checked employing kinetic and thermodynamic results. This treatment reveals the possibility of obtaining the equations describing the different types of catalysis (homogeneous, microheterogeneous, heterogeneous and electrocatalysis) from a common starting point, the Brönsted equation. This circumstance indicates that the distinction between these different types of catalysis, although useful, is not a radical question.

## 3. Microheterogeneous Catalysis with Participation of Ground-State Reactants

### 3.1. General

In this section representative examples of microheterogeneous catalysis will be considered. Here we limit ourselves to the cases in which reactants are in their ground states. The case in which excited states of the reactants participates will be considered in the next section of the review.

The cause for this separation relies in the fact that application of treatments developed in Section 2, implies that the binding of the reactant and catalyst (Equation 1) must be at equilibrium. This requirement is generally accomplished by thermal (ground-state) processes, but in the case of photochemical (excited state) reactions, the possibility of reaching the equilibrium in Equation 1 depends on the lifetime of the excited state and on the rates of the forward and reverse processes in this equation, in such a way that the equilibrium condition does not always hold.

This section has been divided into four subsections corresponding to the cases, in which the catalysts are: (i) micelles (both direct and reverse); (ii) polymers; (iii) saturable receptors, such as cyclodextrins and related systems and (iv) complex systems. Although there is no essential differences between receptors, in the sense that the treatment developed in the previous section can be applied in all of these cases, they have characteristics that can produce some differences. Thus, in the case of saturable receptors, the number of guests bound to a given microcatalyst is generally small and well defined (one or two in some cases). This produces some differences from micelles because, although these kinds of receptors are definitively saturable, given the small concentrations of reactants generally employed in relation to the number of binding sites on the micelles, they behave as practically unsaturable receptors. 

Moreover, reactions between two reactants bound to different cyclodextrins cannot be excluded. On the contrary, the reaction between two reactants bound to different charged micelles is impeded by the repulsive micelle-micelle (or polymer-polymer) interaction. According to this, bound reactants must be on the same micelle. This implies that, for a bimolecular reaction in the presence of micelles there is only two possible reaction paths:

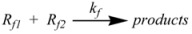
(60a)

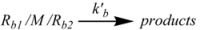
(60b)
whereas in the case of cyclodextrins there are four possible reaction paths:

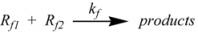
(61a)

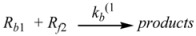
(61b)

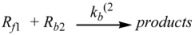
(61c)

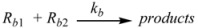
(61d)


This, of course, produces different equations giving the experimental rate constants, *k_obs_*: from the arguments given in Section 2 it is possible to show that, in the micellar case, *k_obs_* is given by [39]:

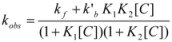
(62)
where *k_f _*is the rate constant for reaction 60a and 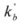 the rate constant for the unimolecular [40,41] process 60b. *K_1_* and *K_2_* represents the binding constants of the reactants to the micelles.

In the case of cyclodextrins (and related systems) Equations 61a**–**d produce the following equation for *k_obs _*[39]:

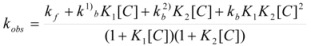
(63)


Moreover, in the case of saturable receptors, the binding constant is a true constant, that is, independent of the concentrations of receptors and reactants. On the contrary, the binding constants can change, in the case of micelles and polyelectrolytes, due to the condensation of counter-ions [42] or cooperative effects [43]. Of course, the treatment developed in Section 2 is still valid, provided that the influence of these effects is incorporated into the treatment [44]. 

On the other hand, micelles and polyelectrolytes present some differences. Thus, in the case of micelles constituted by a unique surfactant all the sites at the micellar surfaces are equivalent. On the contrary, polymeric materials are frequently heterogeneous in the sense that they show different kinds of binding sites. For example, in the case of DNA it is known that ligands can bind to the polymer through electrostatic interactions with phosphate groups, or in the major or minor grooves, as well as through intercalative binding mode [45,46]. It is clear that the reactivity of ligands that depends on the binding will be different in these different sites. Indeed, as the proportion of occupied sites of each class will be dependent on the relative concentrations of ligand and polymer, the (macroscopic) measured binding constant will be dependent on this proportion. In this regard, the polymer behaves as a mixture of receptors.

### 3.2. Micellar Solutions

Reactivity in normal and reverse micelles has been the objective of a great deal of research activity. Thus, many kinds of reactions such as, oxidation, hydroformylation, carbonylation, dehalogenation, free radical polymerization, enzymatic reactions [47], ligand substitution reactions [48], electron transfer reactions [49] *etc*., have been studied in these kinds of systems.

This interest arises from several reasons such as the possibility of localization of reactants in suitable microenvironments, which enables catalyzing and control of a wide variety of reactions [50]. Thus, for example, microemulsions have been used to induce regioselectivity in organic reactions [47]. On the other hand it is possible to solubilize in micellar solutions reagents that cannot be dissolved in the same bulk solvent. In other cases, interest arises because simple microheterogeneous systems mimic some of the most important characteristics of biological systems. Some studies on reactivity in micellar systems are aimed at learning about the structure of these systems [51]. In relation to the latter kind of study, however, it is important to realize that a rate constant is a macroscopic magnitude (in some sense, if one accepts Transition State Theory, a thermodynamic magnitude). As such, it represents an average value and, consequently, cannot give structural information directly. However, thermodynamic and kinetic data are useful in order to check the structural information obtained from other techniques.

Many (but not all) of the reactions in micellar systems happen, at least partially at the interfaces, in this case, at liquid interfaces. An important aspect of these interfaces is the fact that their properties are not uniform through them. This implies that reactivity at different points of the interfaces can be different. Moreover, this inhomogeneous character implies that the total intermolecular force on a given molecule at the interfacial regions is strongly anisotropic. As a result, certain molecular orientations and localizations may be preferred at the interface [52]. Of course, localization and orientation are specific, that is, they depend on the characteristics of the molecule that is being considered. This gives rise to some problems when one is considering some properties of the interfaces. For example, dielectric permittivity at the micellar interfaces has been deduced sometimes employing some probes and measuring properties of the probes related to the dielectric polarity of their environments [53]. These dielectric data are then used to explain reactivities of reactants different from the probes. However, this would not be correct unless one is secure about the fact that probes and reagents are localized in the same region of the interfaces. This caution must also be considered when one is dealing with electric potentials at the interfaces. 

These potentials are determined by using suitable indicators [54]. In the best of cases, this procedure would give the electric potential at the points where the indicators are placed, which are not necessarily the same as the points where other reactants are localized. In fact, it has been suggested that ions of different charges, but with the same charge sign, are localized on different regions of the DNA/water interface. This different localization arises as a consequence of dielectric saturation effects caused by the high electric field at the interface. This effect would push the ions towards the aqueous phase, but more so to the ion with the higher charge. In other words, the ions will feel different potentials according to their different charges [22].

Coming back to consideration of the reactivity in micellar (direct micelles) solutions, the micellar effect has been interpreted generally using the Pseudophase [24] and related models, such as the Pseudophase Ion Exchange Model [55]. The Pseudophase Model of Menger and Portnoy produces the same equations derived in Section 2 from the Brönsted equation. Thus for a true unimolecular reaction, as established in Section 2, Equation 3 is obtained. This equation still is valid for bimolecular reactions provided that one of the reactants remains mainly in the aqueous pseudophase [56]. Thus, for a reaction:

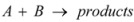
(64)


if only the reactant *B* is partitioned between the micelles and the bulk pseudophase, taking into account the volume of this pseudophase has practically the same value as the total volume of the system, one can write:

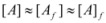
(65)
where:

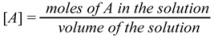
(66a)

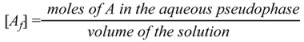
(66b)

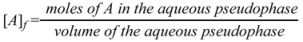
(66c)


In this way, *k_f_* ≈ *k^f^* where *k_f_* has the same meaning as in Equation 3 and *k^f^* is the rate constant in the aqueous pseudophase corresponding to the concentrations of the reactants in this pseudophase. On the other hand, *k_b_* is related to the true (second order) rate constant of the catalyzed reaction, *k^b^*, by:

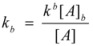
(67)
(notice that according to Equation 65 [*A*]*_b_* << [*A*]*_f_* and [*A_b_*] << [*A_f_*]). Equation 67 can be written as:

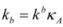
(68)
where:

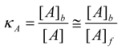
(69)


Of course, *κ_A_* has a different meaning from *K_A_* (see Equation 1), but they are related:

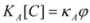
(70)


*φ* being:

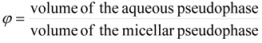
(71)


In other words, for bimolecular reactions, if only one of the reactants is appreciably partitioned between the micellar and aqueous pseudophases, Equation 3 is formally valid. In this equation *k_f_* has the same meaning as that of unimolecular reactions, but the meaning of *k_b_* is somewhat different, as given by Equation 68.

In the case of bimolecular reactions, when the two reactants are partitioned between the micellar and aqueous pseudophases, Equation 3 cannot be applied. Instead Equation 62 must be used. This equation reveals that, in this case, the effect of the catalyst comes, at least in part, from a concentration effect, because local concentration of the reactants is increased. On the other hand, if the intrinsic reactivity at the micellar pseudophase is greater than in the aqueous (or bulk) pseudophase, an additional increase in rate will be observed. Equation 62 implies that *k_obs_* will go through a maximum when [*C*] is changed. Thus, for small concentrations of *C*, in such a way that K_1_[*C*] and K_2_[*C*] << 1, *k_obs_* will depend linearly on this concentration:

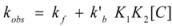
(72)


However, at big enough concentrations of *C*, the quadratic term in Equation 62 will dominate, thus producing a diminution of *k_obs_*. This behavior reflects the fact that when [*C*] is small there is a high probability of finding two reactants on the same micelle, in such a way that the concentration effect is operative. On the other hand, when the concentration of the catalyst is big enough, the probability of finding two reactants on the same micelle is low, and the concentration effect disappears.

It is interesting to note the this behavior was predicted theoretically by Scatchard [27], for salt effects on reactions between ions of the same charge sign, starting from the Brönsted equation and employing the Mayer’s treatment [57] of ionic solutions for the calculation of the activity coefficient of reactants and the transition state. This fact reveals that there are no essential differences between the catalytic effects of salts and micelles on ionic reactions [23].

Equations 3 and 62 have been checked many times in studies on reactivity in micellar solutions. However, several causes can produce deviations from the behavior predicted by these equations. In particular, for charged reactants, the binding constants (Equation 1) change when the concentration of charged micelles does so. This is a consequence of the fact that when the concentration of micelles increases, the association degree of the counterions (of the micelles) does so. Obviously, this produces a diminution of the charge of the micelles that, in turn, reduces the electrostatic component of the free energy of binding.

The effects of counterion association (with the micelles) can be incorporated into previous equations in a simple way [42]. Thus, the free energy corresponding to *K* (Equation 1) can be written, without any lost of generality, as the sum of two contributions: a nonelectrostatic contribution, ∆*G_nel_*, and an electrostatic contribution ∆*G_el_*, dependent on the electrostatic field at the interface, that is, on the degree of association with the counterions:

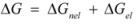
(73)


The electrostatic contribution can be expressed as:

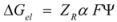
(74)
where *Z_R_* is the charge of the reactant and Ψ the electrostatic potential measured with some probe, generally an indicator. Parameter *α* takes into account that the locations of the indicator and the reactant can be different, as previously mentioned. If one defines:

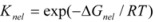
(75)


It is obvious that:

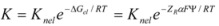
(76)


This procedure for the incorporation of the effects that counterion binding has on the changes in reactivity, has been checked several times by us [42,44,58]. 

Separation of the electrostatic and non-electrostatic components of the free energy of binding can be accomplished using de Lippard’s equation [59]. According to this equation, in the presence of an added salt, log *K_el_* is proportional to **-** log[*X*], *X* being the counterion of the micelles. Thus:

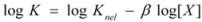
(77)


Consequently, a plot of log *K* *vs*. log[*X*] gives, as the intercep, log *K_nel_*. Once *K_nel_* is known, *K_el_*, for each salt concentration can be obtained as:

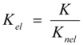
(78)


In this way, we have determined the electrostatic and non-electrostatic component of the free energy of binding of [Ru(NH_3_)_5_Pz]^2+^ to gold nanoparticles [60] and DNA [22,61]. The effects of the changes in the composition of the interface have also been focused from other points of view. Thus as mentioned previously Quina and Chaimovich [55] developed the Ion Exchange Pseudophase Model by applying to the micellar catalysis a treatment analogous to the classical treatment of ion-exchange resins. They considered the case of a micellar solution formed from the detergent *DY* where a salt *BX* of the reactive ion, *X^−^*, is present. According to the authors, *X^−^* occupies a site at the interface through an ion exchange process:

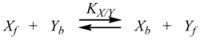
(79)
where:

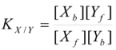
(80)


They assumed that: (1) The distribution of aggregate sizes can be represented in terms of the most probable aggregation number, 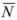; (2) The ion-ion and ion head-group interaction are non-cooperative, in such a way that ion exchange rates are dependent only on the number of ions in a given aggregate and the concentrations of free ions in the bulk; (3) The degrees of ionization *α* of the individual micellar species *MY_i_X_i_* are the same independently of the* i* and *j* values. If *m* is the total number of occupied sites at the micellar surface:

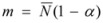
(81)


(4) The ion-exchange processes are rapid relative to the lifetime of micelles; (5) The activities of the different micellar and free species present are treatable in terms of their analytical concentrations. An additional assumption is that *K_X/Y_*, *α* and the cmc are independent of the total detergent concentration [55]. Under these assumptions the following equations hold for the case in which *BY* is added to the solution:

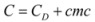
(82a)

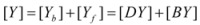
(82b)

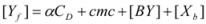
(82c)

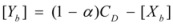
(82d)

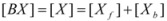
(82e)


From these equations and Equation 80 it follows that:

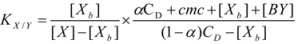
(83)


This equation may be resolved for [*X_b_*] to give:

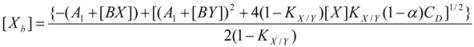
(84a)

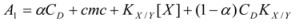
(84b)


Once [*X_b_*] is known, [*X_f_*] can be calculated from:

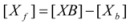
(85)
in such a way that the observed rate constant would be given by:

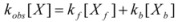
(86)


The authors also considered more complicated situations such as the effect of the addition of a salt *ZB*, the case in which the micellar system remains buffered ,as well as the reaction of *X* with an uncharged substrate, *S*, (when [*X*] >> [*S*]) whose binding to the micelle is described by a binding constant *K_s_*.

The problem that we are considering has been also envisaged by Rodenas *et al*. [62,63]. They employed a treatment based on a cell model of the solution. In this model the micellar solution is divided into cells, each of them containing a micelle and the amount of water and electrolyte given by the whole concentration of the particular systems. The cells and the micelles are considered to be spherical, with radius *r_c_* and *r_m_*, respectively. The micellar charge is considered to be uniformly distributed over the micellar surface. The distribution of ions around the micelles is calculated from the non-linearized Poisson-Boltzmann equation. This equation under spherical symmetry conditions is given by:

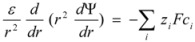
(87)
where *ε* represents the permittivity of the solution, and *z_i_* and *c_i_* the charges and concentrations of the ions present in the solution. By integration of this equation one can obtain the concentration of the reactive ion(s) close to the micellar surface for different concentrations of supporting electrolytes.

In the previous paragraphs we have considered changes in the binding constants of the reactants to the micelles caused by counterion condensation. There are other possible causes for a change in *K*. Thus, this parameter can change as a consequence of a change in the micellar shape. Obviously a change in the shape of the micelles implies a change in the surface curvature and thus in the density of charge in the micelles. This would imply a change in the electrostatic component of the free energy of binding of charged substrates (see Equations 73-76). This possibility of change of the binding constant has kinetic consequences, as shown by Moya *et al*. [64,65]. These authors showed that the change in the micellar shape implies changes in the dissociation degree of the counterions, aggregation number, water content in the interfacial region and local polarity [66]. These changes, as previously mentioned, influence the binding constant, as well as local reactivity. Thus, according to the authors, and due to these changes, an increase in the reactivity is observed, for the reactions of methyl 4-nitrobenzene-sulfonate and methyl naphtalene-2-sulfonate with bromide ion, as a consequence of the sphere to rod transition in solution containing gemini surfactants [64], as well as in solution containing mixed single chain and gemini surfactants.

Another cause of failure of the simple equation derived from the Brönsted equation or from the Pseudophase Model has been observed for reactions between ions of opposite charge sign in micellar solutions [67]. In this case, only one of the reactant ions will be associated to the micelles. The other reactant will remain in the bulk pseudophase. If the charge of the reactant located in the bulk pseudophase is high and/or its concentration (relatively) high and/or the micellar concentration is low, this reactant will compete with the micelles for capturing the other reactant. This will produce a change in *K* (Equation 1) that will depend on the concentration of the reactant non-associated to the micelle. Thus, consider the reaction between two oppositely charged reactants *A* and *B*, and suppose that only *A* is associated with the micelles, according to Equation 1. Now suppose that *A* and *B* can also be associated forming ion pairs *A*/*B*. Reactant *A* can now be in three states: free, bound to the micelles, *A_B_*, and bound to *B*, *A*/*B*. Thus, if one defines:

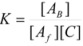
(88a)

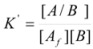
(88b)

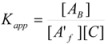
(88c)
where *A′_f_* represents the total concentration of *A* which is not bound to the micelles, that is:

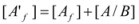
(89)


The apparent binding constant, *K_app_*, will be given by:

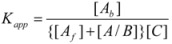
(90)
or:

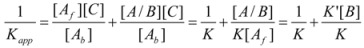
(91)


That is:

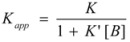
(92)


According to this equation, the value of the effective binding constant to the micelles to be used in Equation 3 will be* K_app_*, that is, dependent on the concentration of *B*. Notice that if the concentration of *B* is not in great excess over the concentration of *A*, its value will change along the reaction time, this producing a deviation of the simple behavior predicted by Equation 3. On the other hand, the binding constant for reactant *A* to a given kind of micelle, determined from kinetic data corresponding to reactions with two different reactants *B* and *C*, having different values of *K′*, will be different [67]. 

An interesting point of view in relation to micellar catalysis is due to Piszkiewicz [68,69,70]. The starting point for Piszkiewicz was the fact that micellar catalysis has been considered frequently as a model for enzyme catalyzed processes. This fact comes from the circumstance that there are important similarities between enzymes and micelles: their structures are similar in that they have hydrophobic cores with polar groups on their surfaces. On the other hand, the structures of micelles are disrupted by common protein denaturing agents such as urea and guanidinium salts. Moreover, both catalytic micelles and enzymes bind substrates in a noncovalent manner and, in some cases, the kinetic of micellar catalysis may be saturated by the substrate, as is the case in enzymatic reactions [69]. Finally, the kinetics of micelle catalyzed reactions shows, sometimes, similarities to the kinetics of processes catalyzed by many regulatory enzymes in the sense that they present positive homotropic interactions (positive cooperativity) [68].

Taking into account these facts, Piszkiewicz proposed a model of micellar catalysis different from the classical Pseudophase Model. In this model it is postulated that the reactant, *R*, and surfactant molecules, *T*, aggregate to form supramolecular *T_n_R* complexes, according to:

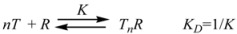
(93a)

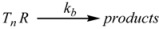
(93b)

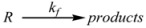
(93c)


According to this, *k_obs_* is given by:

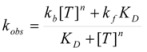
(94)


The reaction mechanism in Equation 93 is similar to the mechanism proposed by Hill [71] to describe the sigmoidal dependence of rate on substrate concentration in enzymatic reactions:

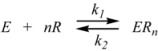
(95a)

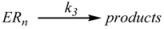
(95b)


According to this mechanism, the reaction rate, *v*, would be given by:

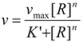
(96a)

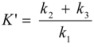
(96b)


Equations 94 and 96a can be rearranged to give, respectively:

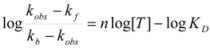
(97a)
and:

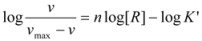
(97b)


If in Equation 97a *k_f_* << *k_obs_* (that is if the non-catalyzed reaction is not significant) it results in:

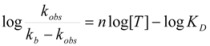
(98)
which is formally identical to Equation 97b.

Previous equations for micellar catalysis describe the sigmoidal variations of *k_obs_* *vs*. [*T*] observed at low concentrations of surfactants in some micelle-catalyzed reactions [68,69,70]. An interesting point in this treatment is that *n* is much lower than the aggregation number normally found for micelles. This has been considered as a manifestation of catalysis by pre-micellar structures. This possibility has been treated by us [72,73].

On the other hand, Biresaw and Bunton [74] have also considered the catalytic effect of premicellar aggregates. Their model also explains the sigmoidal dependence of the observed rate constant on [*T*], at low values of this concentration (see also reference [73]). This treatment is based on the following assumptions: (1) the model is applied to short chain surfactants that forms polydisperse aggregates (instead of micelles) in such a way that they do not exhibit a critical micelle concentration; (2) This aggregation may be assisted by cooperative interaction with the reactant (which experiences the catalyzed reaction), as in Equation 95a; (3) The aggregates are formed (at least formally) via a stepwise-self-association process, consistent with the absence of a critical micellar concentration; (4) The distribution of the reactant between the continuous phase (water) and the aggregate with *q* monomers, *T_α_*, for *q* ≥ 2 is given by an equilibrium constant *K′_α_*; (5) The rate constant for the reactant bound to *T_α_* is *k′_α_*. Two simplifying assumptions are: (6) *k′_α_* = *k_b_*, independently of *q* and (7) *K′_α_* = *qK_s_*, *K_s_* being the intrinsic substrate binding constant expressed per monomer concentration. According to these assumptions:

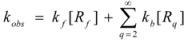
(99)
where *R_q_* represents the reactant bound to *T_q_*. The concentration of *R_q_*, according to previous assumptions, is:

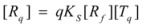
(100)
and thus:

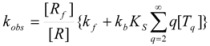
(101)


If [*R_b_*] is the total concentration of the bound reactant,

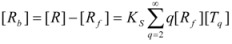
(102)


So:

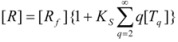
(103)
and consequently:

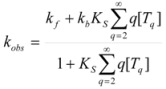
(104)


This equation can be simplified assuming that stepwise association constants corresponding to processes:

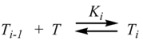
(105)
are independent of the value of *i*, that is:

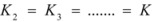
(106)


Under this circumstance, results for *k_obs_*:

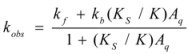
(107a)

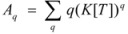
(107b)


This equation describes well the catalytic effect of the aggregates on unimolecular reactions, of the type described by the Piszkiewicz model, with reasonable values of the parameters appearing in the equations [74]. As, in the Piszkiewicz model, a sigmoidal dependence of *k_obs_* of [*T*] is predicted by this treatment. However, maximum values of *q* are greater than the values of *n* found in the Piszkiewicz treatment. 

All the previous considerations and treatments can be applied to both, direct and reverse micelles. The latter, however, have some special characteristics that produce some differences with the direct micelles. Thus, in reverse micelles most of the water present in the system is confined water, particularly, at low values of the molar ratio *w* = [*H_2_O*]/[*surfactant*] [75]. This water has very different properties from bulk water (see Introduction section) which influences reactivity. The unique feature of the entrapped water can be exploited fruitfully in various applications in biotechnology [17,18]. It is interesting to note that in the last few years the interest of the studies (including reactivity studies) in reverse micelles is more to learn about the characteristics of confined water than to learn about the catalytic properties of reverse micelles. However, at least in some cases, reverse micellar systems can become more complex that systems containing direct micelles. Solutions of direct micelles are considered biphasic (or pseudobiphasic) systems. In the case of reverse micelles one can conceive the possibility of reactions that happen simultaneously at the interface (the surfactant region) the confined water region and the oil or continuous pseudophase. In this way, the system becomes a pseudo-triphasic system.

For these reasons, some specific treatments have been developed for reactions in reverse micelles. Thus, in reference [44] the oxidation of [Ru(NH_3_)Pz]^2+^ by S_2_O_8_^2−^ (Pz = pyrazine) in reverse micelles was studied. The studies were conducted at different *w* values and at constant concentrations of the reactants in the aqueous pseudophase. Given the ionic character of the reactants it was considered that they were absent in the oil pseudophase. In order to explain the reactivity trends, it was supposed that one of the reactants (the ruthenium complex) was adsorbed at the interface, the other remaining mainly in the aqueous pseudophase. For a given value of the molar ratio, *w*, assuming that all the surfactant and water molecules are incorporated into the micelles, if *V* is the volume of the water pool it is possible to write:

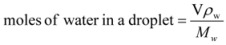
(108)


*M_w_* and *ρ_w_* being the molar mass and the density of water. From the value of the molar ratio it follows that:

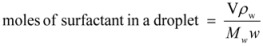
(109)


This number is equal (or proportional) to the number of binding sites in a droplet. Thus:

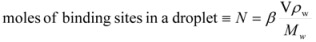
(110)
where *β* is a proportionality constant, which takes into account the possibility of a substrate binding to more than one polar head of surfactant.

Considering the microheterogeneous equilibrium:

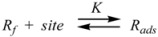
(111)


If one assumes a Langmuir type of the adsorption process, one can write:

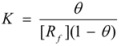
(112)


*θ* being the fraction of occupied sites

On the other hand:

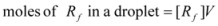
(113)


and:

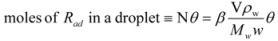
(114)
and, evidently:

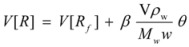
(115)


Under the working conditions in this work, *θ* << 1 (even if the ruthenium complex is totally adsorbed). Thus:

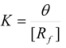
(116)


in such a way that:

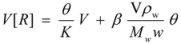
(117)


From this equation,

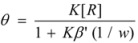
(118a)

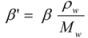
(118b)
and using Equation 34 one finds:

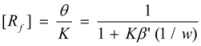
(119)


From Equations 118a and 119 it follows that the observed (*second order*) rate constant is given by:

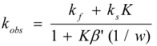
(120)


*k_s_* being the rate constant for the surface reaction, and *k_f_* the corresponding parameter for the water pool.

As mentioned previously, microemulsions can behave in some cases as (pseudo) triphasic systems. For these cases, the most direct way to find the equation giving the observed rate constant is to employ the formulation developed in Section 2, that is, the formulation based on the Brönsted equation. Using this approach it can be shown [76], for the general case of a system containing a continuous phase, I, and *n-1* dispersed phases in which a reaction is possible, that the observed rate constant, if one takes phase I as reference, is given by:

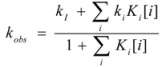
(121)


This equation for the particular case of a microemulsion oil/water/surfactant, if one takes as reference the oil phase gives:

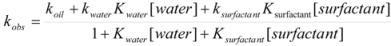
(122)
where *K_water_* and *K_surfactant_* are defined as in Equation 1.

Other treatments for particular cases have been developed. Thus, Garcia-Rios *et al*. considered the cases of thermodynamics and kinetics of complexation of Ni^2+^ and Co^2+^ by PADA (pyridine-2-azo-*p*-dimethylaniline) in AOT/isooctane/water microemulsions [77,78]. The authors considered microemulsions to be made up of three different microenvironments: a continuous phase formed by the alkane, a disperse phase formed by the aqueous micro-droplets and a surfactant film that separates the aqueous phase from the alkane phase. The authors, from the variation of the absorption spectrum of the ligand (PADA), concluded that it is distributed among the three pseudophases. The distribution equilibrium constants 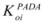 (*oi* = oil/interface) and 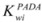 (*wi* = water/interface) are defined as:

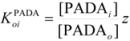
(123a)

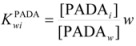
(123b)
where *w* has its habitual meaning and *z* is the molar ratio [*isooctane*] /[*AOT*]. In fact 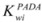 is high, in such a way that the ligand PADA can be considered distributed only between the oil and surfactant pseudophases. On the other hand, the Ni^2+^ and Co^2+ ^ions are present only in the aqueous pseudophase and at the interface. This distribution is viewed as an ion exchange process between the counterions of the surfactant, Na^+^, and the M^2+^ cations, and quantitatively described by an equilibrium constant 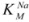 given by:

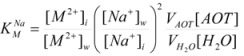
(124)


From previous considerations it is obvious that the complexation reaction can happens only at the interface, according to:

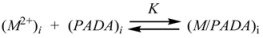
(125)


*K* in previous equation is related to the observed equilibrium constant *K_obs_* (referred to the concentrations of the reactant in the system) by:

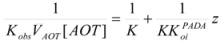
(126)


According to this equation, the plot of 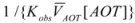 *vs*. *z* should be linear. From the slope and intercept of this plot the authors were able to obtain *K* and 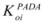. An interesting result is that *K* depends on *w*, that is, the structure of the interface depends on the water contents of the system [77].

Using the same reaction scheme, the authors were able to relate the observed rate constant, *k_obs_*, to the rate constant for the formation/dissociation rate constants at the interface:

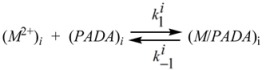
(127)

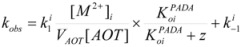
(128)


From this equation the authors derived the values of 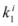 and 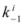. These values, as *K*, depend on the water contents of the microemulsion. In fact 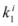 changes much more than 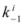 in such a way that variations of 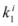 control the changes in the equilibrium constant.

To conclude this section one can say that two state models describe well the variations in reactivity in micellar systems. These models, in fact, are particular cases of the Brönsted formulation: they can be deduced from it. This equation, indeed, permits generalization to multiphasic systems. It is important to realize that final equations, in any case, depend on parameters defined as equilibrium or distribution constant. However, these parameters are not true constants, in the sense that they depend on the structure of the system (micelles) that can change if one works in a wide range of conditions. Several examples of the causes that produce changes in these “constants” have been considered, as well as the procedures to take them into account.

### 3.3. Polymers and Related Microcatalysts

Polymers can act as receptors with some similarities to micelles. Thus, the reactivity in the presence of DNA, a negatively charged natural polymer, can be modelled according to the Pseudophase Model. In fact, changes in *k_obs_* for reactions in the presence of DNA are given by Equation 3 (when only one of the reactants participating in the reaction is bound to polymer), or Equation 62 (if the two reactants are bound to DNA) [79]. However, there are some differences between polymer effects and micellar effects. Thus, polymers are, generally speaking, heterogeneous materials in the sense that they show different kind of binding sites. In the case of DNA, for example, some ligands bind to DNA through electrostatic interactions with phosphate groups, whereas others prefer major or minor grooves to bind. Finally, other ligands are intercalated between the base pairs [45,46]. Moreover, polyelectrolytes are more flexible than micelles and, according to the properties of the surrounding medium can suffer conformational changes that in turn produce changes in the binding constants of the ligands (reactants). For example, changes in the ionic strength will produce, changes in the binding constants for reactants having charges of opposite signs to those of the polymer [80,81]. In part, this effect can be ascribed to the screening effect of the ionic clouds of the polymer and ligand. However, this screening effect is not the unique effect of increasing ionic strength. This is clearly demonstrated by the fact that the binding constant of the neutral ligand of pyrene-1-carboxyaldehyde to DNA decreases when the ionic strength of the surrounding medium increases [82].This points to the idea that the conformation of DNA is altered by the salt (probably by the counterions of the salt). In fact, electrolytes can produce conformational changes in DNA dispersed in water by going from a random coil to a helix structure [83]. Neutral cosolutes, like alcohols, also produce changes in the binding constants [82] of ligands to DNA, as do surfactants [84]. In this case, this change is due to the conformal change of DNA from the random-coil to globular structure induced by the surfactant [85]. Obviously, the structural changes can be induced by the ligand (reactant) itself, which would produce a change in the binding constant of the ligand. This circumstance would give rise, at least formally, to cooperative effects [86]. Fortunately, equations of the Pseudophase Model are flexible enough to incorporate the above mentioned effect. It suffices to consider that the binding constants, in this case, depend on the concentration of the species in the reaction media producing the alteration on the DNA structure. In fact, the reactant(s) can induce a change in the DNA structure. In this case, the binding constant will be dependent on the adsorption degree of the reactant(s), that, in turn, will be dependent on the relation of the concentrations of the catalyst (DNA) and the reactant. If, as generally happens, the concentration of the reactant is fixed and the DNA concentration is changed, one will observe a dependence of the binding constant on the concentration of the catalyst. If the binding constant decreases when the concentration of DNA increases, this will be the consequence of a positive cooperativity. On the contrary, if the binding constant increases when the DNA concentration does so, the system shows a negative cooperativity [45].

Unless for the existence of cooperativity, the behavior of DNA as catalyst is quite similar to the effect of charged micelles, as are the effects of nanoparticles [60] and charged dendrimers [44], that is, changes in *k_obs_* can be described by Equation 3 or 62 with the binding constant appearing in these equations being true constants or parameters which depend on the concentration of the catalyst.

As mentioned previously, changes in the ionic strength in the solutions containing DNA influence the strength of binding of charged solutes. These changes follow the Lippard’s equation [59]. In the presence of a sodium salt this equation is:

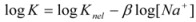
(129)


In this equation *K_nel_* represents the non-electrostatic part of *K* and *β* is a constant dependent on the charge of the ligand. *K_nel_* can be obtained from the (linear) plot of log *k* *vs*. log[*Nα*^+^] and, once the non-electrostatic part of the binding is established, the electrostatic part can be determined (see Equation 78). In turn, one can write:

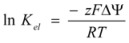
(130)
in such a way that one can determine the value of the difference in the electrostatic potential between the solution and the region close to DNA where the ligand binds to it [22]. Following this approach, the values of ∆Ψ have been estimated using as probes [Ru(NH_3_)_5_Pz]^2+^ and [Co(NH_3_)_5_Pz]^3+^. The values of ∆Ψ for these probes are consistent, which gives support to the procedure.

Another kind of polymer with similar behavior to DNA are polypeptides [87,88]. Sanchez *et al*. employed lysine/glycine polypeptides with different degrees of polymerization. The different peptides produce a decrease in the rate of the reaction between [Co(C_2_O_4_)_3_]^3-^ and [Ru(NH_3_)_5_Pz]^2+^. From these rate changes, the binding constant of the cobalt complex can be assessed. An interesting result from these studies is that the binding free energy, *per monomer* increases with the degree of polymerization. That is, the aggregation of monomers increases the free energy of binding. This increase in the negative free energy of binding represents a driving force for the polymerization process. Thus for *n* (number of monomer) 7, 14 and 21 the differences in the free energies of polymerization in the presence and in the absence of the cobalt complex are respectively. −9.5, −16.7 and −18.5 kJ/mol.

As to the effects of dendrimers on chemical reactivity, they behave as do DNA and other polymers such as polypeptides. Thus, in the presence of negatively charged dendrimers, the changes in the rate constant of the oxidation of [Ru(NH_3_)_5_Pz]^2+^ by 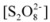 (only the ruthenium complex binds to dendrimers) follow the expected trend according to the Pseudophase Model [44]. The same behavior is found when the oxidant is [Co(C_2_O_4_)_3_]^3-^, thus confirming that only the ruthenium complex binds to the dendrimers [89]. 

The effects of dendrimers on ligand substitution reaction have also been studied [15,90]. As in other kinds of reactions, the Pseudophase Model also holds for this type of process. It is interesting to note that in reference [90] the authors studied the influence of the dendrimers at several values of the ionic strength. By following the same procedure as in the case of DNA, the electrostatic and nonelectrostatic components of the binding constant were separated.

Nanoparticles exhibit similar behavior to polymers and dendrimers. Thus the kinetics of the oxidation of [Ru(NH_3_)_5_Pz]^2+^ by 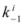 has been studied in the presence of gold nanoparticles caped with *N*-(2-mercaptopropyonyl)glycine [60]. The kinetics, when the concentration of nanoparticles is changed, follows the trend corresponding to the equation of the Pseudophase Model with a high binding constant for the union of the ruthenium complex to the nanoparticles. According to the authors there is some anticooperative character in this union. However, this anticooperativity can be only formal in the sense that it could be due to saturation effects at the surface of the catalyst. The authors worked at different ionic strength in such a way that they were able to separate the electrostatic and non-electrostatic components of the binding free energy. According to their results, the electrostatic component is the main driving force for the union.

The effects of AlOOH nanoparticles as microcatalyst have been studied by Sánchez *et al*. [91]. The study was performed at different ionic strength using as a probe the kinetics of the oxidation reaction of [Ru(NH_3_)_5_Pz]^2+^ by 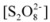 at pH = 5.4. In this case, it is the anion the ligand that binds to the nanoparticle. The rate of reaction changes by a factor of about ten in the presence of nanoparticles; this change decreasing as the ionic strength increases. Again, as in the case of gold nanoparticles, the main component of the free energy of binding is the electrostatic part.

So, it can be said that polymers and related receptors behave in a similar way to that of micelles, although in some cases they can exhibit a somewhat more complex behavior due to the conformational changes that the process of reactant binding can induce in the polymer. But even in these cases, the Pseudophase Model can still be applied, although the binding “constant” would be variable, that is, it would dependent on the [receptor]/[ligand] ratio.

### 3.4. Solutions Containing Saturable Receptors

Now the case of microheterogeneous catalysis produced by receptors such as cyclodextrins and similar receptors will be considered. First of all, it is important to realize that there are no significant differences between these kinds of receptors and, for example, micelles. Thus, the Pseudophase Model can be applied to the interpretation of data in, for example, cyclodextrins solutions [44]. However, we have considered pertinent the inclusion of a separate section for these kinds of receptors, that is, of receptors in which the number of guest molecules is small and well defined (one, or two, in some cases) [92]. Moreover, as mentioned previously, reactions between two reactants bound to different receptors are possible, in such a way that Equation 63 would apply. 

However, for low concentrations of the receptor and/or big binding constant to saturable receptor, application of equations such as Equations 3 or 63 must be done with caution: strictly speaking, in these equations [*C*] represents the concentration of free catalyst. When this concentration is big enough, it can be taken as the total concentration of *C*. However, some saturable receptors (as for example β-CD) present a low solubility in such a way that the concentration of a free catalyst can be quite different from the total concentration. Under these circumstances, if one uses equation 3 with [*C*] = total concentration of the catalyst, a variable (apparent) binding constant will be obtained, and, for a fixed ligand concentration this constant will increase when the concentration of *C* does so, in such a way that the system would behave, formally, as a system in which the ligand/catalyst binding was anti-cooperative. Thus, it can easily be shown that the apparent binding constant *K_app_* defined as:

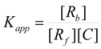
(131)
is related to the true binding constant, *K*, by:

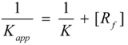
(132)
in such a way that *K_app_* will only be equal to *K* when [*Rf*] = 0, that is for high enough concentrations of *C* for producing a negligible concentration of free reactant. 

Considering now the effects of cyclodextrins (and related systems) on the reactivity of bound reactants, one can find examples in which this reactivity increases or decreases in relation to free ligands. In fact, sometimes small modifications of the receptor can change the effect of binding on the reactivity. Thus, β-CD decreases the rate of oxidation of [Fe(CN)_5_4phepy]^3-^ by [Co(NH_3_)_4_(H_2_O)_2_]^3+^ whereas the rate of this reaction increases in the presence of 2-hydroxypropyl*-* β-CD. On the other hand, for a given cyclodextrin, changes in reactivity can also be tuned through the addition to the reaction medium of substances, such as co-solvents or salts, which change the binding constants of the reactants [93,94,95,96,97,98].

Cyclodextrins are by far the most studied saturable receptors. Thus, their effects on a variety of processes have been studied. The effects of cyclodextrins on electron transfer processes have been considered above. The effects of these kinds of receptors on biomimetic reactions have been reviewed [99] as well as studies of cyclodextrin effects on organic reactions [100,101,102,103,104,105,106], redox reactions [107,108,109], ligand substitution reactions [110], *etc.*, in such a way that the effects of this kind of catalyst on practically any type of reaction have been studied.

Other saturable receptors have been less studied than cyclodextrins. Among these receptors calixarenes might be the most employed. These are macrocyclic compounds obtained through condensation of phenols with formaldehyde. They can act as hosts for ions and small neutral molecules. The selectivity and efficiency of the binding process depend on the ring size, on the nature of the attached groups (to the phenol component), and on the stereochemical conformation of these versatile macrocyclic molecules, that can generate a large number of conformations. This conformational freedom is a key factor in the stabilization of the particular host-guest interactions between calixarenes and their guests [111]. This conformational flexibility increases for the larger rings.

Calixarenes have been employed for alkali-metal cations transport through hydrophobic organic phases and the binding constant of the complexes have been measured [112], as well as the influence of additives on the complexation by calixarenes [104,111].

Supramolecular structures formed by the condensation of two or more calixarene units were prepared and their properties studied. Casnati *et al*. [113] have reviewed the synthesis and properties of these supramolecular structures. They studied specifically the structures formed by calixarenes endowed with α-amino acids. Curiously, *N*-linked peptidocalix[4]arenes show properties quite different from those of the corresponding *C*-linked derivatives. The latter show a tendency to form self-assembled nanotubes in the solid state. An interesting property of these macrostructures is that hydrogen bonding, donor and acceptor groups of the aminoacid residues and the cavity of one calyx [4]arenes act cooperatively in guest binding in nonpolar solvents but not in water, where hydrophobic interactions dominate.

The number of studies on the influence of calixarenes on the reactivity of the guest is relatively small compared to the corresponding studies on cyclodextrins. Among these studies Lopez-Cornejo *et al*. [114] studied the influence of p-sulfonatocalix[n]arenes (n = 4, 6, 8) on the kinetics of the oxidation of [Ru(NH_3_)_5_Pz]^2+^ by [Co(C_2_O_4_)_3_]^3-^. According to the authors, only the ruthenium complex interacts with the receptor. The kinetic results can be rationalized using as a starting point of the Pseudophase Model. The effect of salts on the complexation process and on reactivity was also studied. The authors concluded that the effect of the receptors on the kinetics depends on the flexibility of the host.

Lopez-Cornejo *et al*. [115] have studied the effects of 4-sulfocalixarene on the kinetic of the oxidation of [Ru(NH_3_)_5_Pz]^2+^ by [Co(C_2_O_4_)_3_]^3-^ and 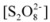. In this case the complex [Ru(NH_3_)_5_Pz]^2+^ /calixarene has a 1:2 stoichiometry. When salt (NaCl) is added, a mixture of complexes with 1:1 and 1:2 stoichiometries is formed. Again, the Pseudophase Model can be used for rationalization of the results.

To conclude this section, one can say that saturable receptors do not present special differences in relation to the nonsaturable in the sense that the Pseudophase Model can be employed in the rationalization of the effects of these receptors on reactivity.

### 3.5. Complex Systems

In this section the effects of catalyst mixtures will be considered. Obviously, these mixtures produce more complex catalytic effects than those of simple catalysts considered previously. However, they offer the possibility of fine tuning of the catalytic effects. Moreover, they are also interesting as model for complex polytopic single catalysts.

In a mixture of catalysts two possibilities arise. The first one is that the two (or more) catalysts do not interact. This case implies that the reactant will interact with the two catalysts independently. A situation of this type arises, for example, for a mixture of neutral cyclodextrins. This system has been investigated by some of the present authors who studied the effects of mixtures of methyl-β-cyclodextrin (MβCD) and 2-hydroxypropyl-β-cyclodextrin (HβCD) on the reaction of oxidation of [Fe(CN)_5_(4-phepy)]^3- ^by [Co(NH_3_)_4_(H_2_O)_2_]^3+^[76]. In this case, only the iron complex interacts with the cyclodextrins (by encapsulation of the 4-phepy ligand). For this reaction, when only one catalyst is present, an increase in the rate of reaction is observed, more marked for MβCD. As expected, the effect of the catalysts follows the simple law represented by Equation 3. In the case of mixtures of cyclodextrins, the experiments were performed for a fixed concentration of MβCD and a variable concentration of HβCD. For [MβCD] = 2.1 × 10^−3^ mol·dm^-3^, the effect of increasing [HβCD] is the expected one: an increase in the reaction rate. However, for [MβCD] = 3.8 × 10^−3^ mol·dm^−3^ no effect of [HβCD] was observed. Finally, if [MβCD] = 2.5 × 10^-2^ mol·dm^−3^, an increase of [HβCD] produces a diminution of the reaction rate. In all cases, the results can be described by the equation:

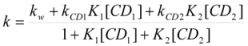
(133)


According to this equation, the observed behavior is the result of a competition of the two catalysts for the iron complex.

The second possibility for a mixture of catalysts corresponds to the case in which the catalysts interact among themselves. This happens, for example, in mixtures of surfactants (neutral + charged or anionic+cationic surfactants). These mixtures produce micelles in which the surface charge density can be controlled by changing the proportion of the surfactants in the mixture. This topic has been studied by Moya *et al.* [65,116,117,118,119,120] Thus, in reference [116] the authors studied the effects on the base hydrolysis of 1,1,1-trichloro-2,2-bis(*p*-chlorophenyl)ethane and 2-(*p*-nitrophenyl)ethyl bromide of mixed micelles of tetradecyltrimethylammonium bromide and penthanol. The presence of the alcohol produces a change in the ionization degree of the micelles as well as a change in the cmc. In all cases the results can be interpreted using the Pseudophase Ion Exchange Model [55].

The spontaneous hydrolysis of phenyl chloroformate in the mixed micelles has been studied by Moya *et al.* [117]. An interesting result is that the effect of the charge (of micelles) is not the only factor responsible for the variation in the reaction rate: depletion of water in the interface region, as well as the structural changes in this region also influences reactivity. Among these factors, in complex systems constituted by surfactants and alcohols, are: an increase in the volume of the micellar interfacial region upon increasing alcohol concentration and a decrease of the polarity in this region caused by the presence of alcohol [118]. The applicability of the Pseudophase Model in solutions containing mixtures of surfactants (CTACl and TX-100) has been shown by Moya *et al*. [119] using as a probe the reaction between methyl-4-nitrobenzenesulfonate and Cl^-^. In this mixture, according to the authors, the neutral surfactant has little influence on the characteristics of the interfacial region where the reaction takes place.

In a kinetic and structural study in solutions containing SDS/Tween 20 and SDS/SB3-12 mixtures of surfactants, Moya* et al.* [120] showed that in these mixtures two types of micellar aggregates are formed upon increasing surfactant concentration. However, kinetic data do not show discontinuity, which was interpreted by the authors as a consequence of the fact that the interfacial region is similar in the two kinds of aggregates. Morphological transitions in aggregates formed in solutions containing mixed surfactants have also been observed in mixtures of single chain and gemini surfactants [70]. These transitions were reflected in kinetic data.

The systems constituted by mixed surfactants have interesting properties outside the catalysis field. Thus, micelles constituted by mixtures of anionic and cationic surfactants (catanionic micelles) have been used as a model to mimic biological membranes in the presence of anaesthetic alcohols [121]. A remarkable analogy was found between the influence of the alcohols on the solubility patterns of the catanionic micelles and on the anaesthesia of tadpoles. According to the authors, the alcohols are incorporated into the biological (lipid) membranes in the anaesthetic process. On the other hand, the changes in solubility of catanionic micelles are also due to the incorporation of alcohol into the micelles. This incorporation, in both cases, requires the presence of a relatively long chain in the alcohols, in such a way that they can enter into the hydrophobic parts of membranes and catanionic micelles.

An interesting complex system constituted by surfactants (SDS and TTABr) and β-cyclodextrin (native β-CD and capitol) has been studied by Garcia-Rio *et al*. [122]. The kinetic effects of such systems were explored using as a probe the hydrolysis of 4-methoxybenzenesulfonyl chloride (MBSC). Micelles and cyclodextrins (separately) influence the hydrolysis in the expected way: kinetic data follow Equation 3 when the micellar or cyclodextrin concentrations are changed. However, in mixed surfactant/CD systems the behavior is more complicated. (In this case, the receptors interact forming inclusion complexes, pseudorotaxanes). The study was performed at three fixed cyclodextrin concentrations and variable surfactants concentrations. The observed rate constant when the surfactant concentration is changed goes through a maximum, in spite of the fact that both, cyclodextrins and surfactants, separately, decrease the rate of hydrolysis. Changes in the rate of the hydrolysis follow equation [122]:

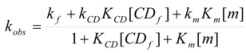
(134)


In this equation *K_CD_* represents the binding constant of the reactant to cyclodextrin and *K_m_* the binding constant to the micelles. [*CD_f_*] represents the concentration of free cyclodextrin, that is, the concentration of this receptor that does not form a pseudorotaxane with the surfactant. On the other hand, *K_m_* is the binding constant of MBSC to micelles. The concentration of free cyclodextrins is calculated by solving the equation:

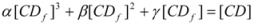
(135)


[*CD*] being the total cyclodextrin concentration and:

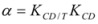
(136a)

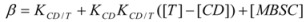
(136b)

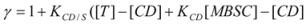
(136c)


In the previous equations [*T*] is the total surfactant concentration and *K_CD/T_* is the binding constant of the surfactant to cyclodextrin to form the pseudorotaxane:

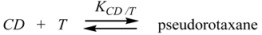
(137)


Interpretation of the results is quite simple: both, cyclodextrins and micelles produce a decrease in the rate of MBSC hydrolysis. So, in the presence of a given amount of cyclodextrin, the rate of reaction is lower than it is in water. When the surfactant is added to the solution, it competes with MBSC for the cyclodextrin. Thus, some MBSC is liberated and reacts more rapidly. When all the cyclodextrin is occupied by the surfactant, the maximum reaction rate is reached. After this point, a new addition of surfactant implies the formation of micelles that decrease the rate of the reaction. It is interesting to note that similar behavior is observed in mixtures of surfactants and DNA [123].

Among the aggregates formed from more than one component, the ones presented by Lee *et al*. [124] are interesting. These aggregates are water soluble toroids with a hydrophobic cavity formed by co-assembly of laterally grafted rod amphiphiles. It is interesting that the toroids are tubularly stacked when a guest (C_60_) is encapsulated in the toroids. For the moment, there are no studies on the influence of these aggregates on reaction rates.

To conclude this section: the possibility of preparing aggregates with more than one component is practically unlimited. These aggregates are of interest because they can be used in many fields, including solar energy conversions (see Section 4.5). In the field of microcatalysis, as we have seen in the examples considered in this section, the door to fine tuning of the reaction rates is opened. 

## 4. Microheterogeneous Catalysis with Participation of Excited States of Reactants

### 4.1. General

The study of photochemical reactions in the presence of receptors that can catalyze them is a long standing matter. In this case, however, given that photochemical reactions are, generally speaking, rapid processes, the interest does not come from the catalytic effect itself. The main interest originated from the objective of finding artificial photosynthetic systems that mimic the natural ones [125]. So, it has been established that “any practical system for solar energy conversion and storage will involve heterogeneous reactions at some stage of the process” [126]. This follows from the need to isolate the primary products of the light-induced charge separation to avoid the rapid recombination of these rich in energy products (back reaction). Obviously, if these products are produced in different phases, the problem of the back reaction can be solved. Thus, consider a photoinduced electron transfer between two neutral reactants, which is produced in a solution containing, for example, negatively charged micelles:

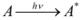
(138)

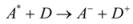
(139)


The reaction products bear different charge signs in such a way that the oxidized donor, D^+^, will have a tendency to go to the negatively charged micelles whereas the negatively charged product, A^-^, would be partially or totally eliminated from the region close to the micelles. This separation of the products would eliminate (or reduce) the back electron transfer:

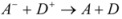
(140)


On the other hand, the presence of receptors in a medium where a photochemical reaction happens, can promote a true catalytic effect giving rise to product selectivity [127], which is of interest in relation to synthetic chemistry.

Photochemical reactions can be influenced by all the effects considered in Section 1 of this review in relation to thermal processes. Moreover, these changes can be detected in many cases because the incorporation of a species into a receptor produces changes in its photochemical properties, such as lifetime of the excited state, emission spectrum, *etc.* These changes for polytopic receptors depend on the kind of site where the excited species is located. This circumstance implies the possibility of using the changes in photophysical properties of bound species (with respect to free species) as probes for investigations of environment properties and characteristics of the receptors. Thus, the dynamics of solvation of bound species has been investigated using fluorescent probes [128]. Fluorescence depolarization studies provide information concerning the mobility of the probe and its orientation (relative to receptor) as well as the mechanical characteristics of the probe environment. In this way, the microviscosity of the region where the probe is solubilized can be estimated [129]. In the field of micellar solutions, the use of fluorescent probes is well documented. Thus, critical micellar concentration, aggregation or the distribution of solutes among the different pseudophases have been studied using fluorescence techniques [130].

An important question in relation to microheteogeneous catalysis, when one of the reactants is in an excited state, is related to the applicability of the models developed in previous sections. These models are based on the assumption of an equilibrium distribution of the reactant between bound and free states (Equation 1). This, of course, implies that the rate of the reaction in which *R* participates is slow in relation to the forward and reverse processes in Equation 1. This introduces the question: are previous models applicable given that the photochemical reactions are generally rapid reactions? Moreover, in the case these equations hold, in the sense that experimental data can be fitted to them, do the equilibrium constants appearing in these equations correspond to ground or to excited states? In relation to these questions, two limiting cases will be considered:

(**1**) In the first case (fast exchange limit) it will be assumed that the kinetics of the interaction of the excited species with the receptors is rapid enough, in the forward and reverse directions, to assume that this process:

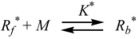
(141)
is at equilibrium.

(**a**) It will be considered, firstly, that the reactant, R^*^(free or bound) can react with a second species (the quencher) *Q*. This reaction is followed by measuring the fluorescence decay of R^*^ after (for example) a laser pulse that produces it:

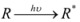
(142)


It can be shown [131] that under these circumstances the observed rate constant for the decay of the emission is given by:

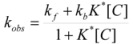
(143)


In Equation 143,

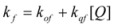
(144a)

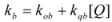
(144b)


*k_oi _*being the spontaneous decay rate constant and *k_qi_* the rate constant corresponding to the quenching reactions (*i* = *f*, *b*).

Thus, one can write:

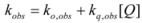
(145)
where:

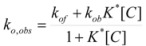
(146a)

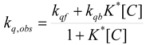
(146b)


Consequently, the spontaneous (*k_o_*_,*obs*_) as well as the quenching (*k_q_*_,*obs*_) rate constants will follow the equations of the Pseudophase Model, but with K^*^, the equilibrium constant corresponding to the partition of the excited species, instead of *K* (see Equation 1).

(**b**) Now suppose that one is performing steady-state measurements, in such a way that the kinetics is monitored making use of the Stern-Volmer equation (and the lifetimes in the absence of the quencher). Under the same circumstance that in the previous case, the observed Stern-Volmer constant is given by [131]:

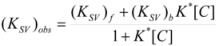
(147)


In this case, the observed Stern-Volmer constant is also consistent with the equation of the Pseudophase Model. However, only if *k_of_ = k_ob_ = k_o _*one can write:

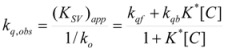
(148)
in such a way that one recovers Equation 146b.

**(2**) In the second case, the slow exchange limit of R^*^, will be considered.

(**a***)* In this limit, the decay of emission intensity (after a laser pulse) will be biexponential [132]:

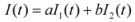
(149)
as corresponding to the fact that, once the fluorophore is excited at *t* = 0, the concentrations of R_f_^*^ and R_b_^*^ correspond to the equilibrium distribution of the ground state and there is no exchange between the excited state populations.

(**b**) In the case of steady fluorescence measurements, it can be shown that [131]:

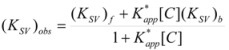
(150)
where:

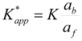
(151a)

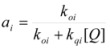
(151b)


Equation 150 is formally identical to the one predicted by the Pseudophase Model. However, 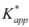 depends on [*Q*] (through *a_f_* and *a_b_*). Thus, unless *k_oi_*>>*k_qi_* (*i* = *f*, *b*), curved Stern-Volmer plots may result in this case.

Previous examples, in which the equation of the Pseudophase Model holds, at least formally, are, in fact, extreme and simplified cases. Apart from the assumptions specifically indicated, in the deduction of previous equations it was assumed that the quencher does not interact with the receptor, in such a way that only one concentration of this species is used.

In the general case, when the kinetics of the union/separation of the reactant (and or the quencher) to the catalyst are coupled to the kinetics of the spontaneous decay and/or quenching process the situation is much more complex. Thus, Yoshida *et al*. [133] obtained the equation of *I_o_*/*I* for the case in which only *R* interacts with the catalyst:

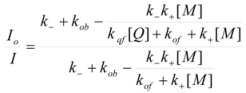
(152)


Previous paragraphs give an idea of the complexity of microheteogeneous catalysis when the reactant is in an excited state. This complexity implies that in order to obtain information on this kind of system one must take care and make a judicious choice of reactant (or the reactant/quencher couple). However, if this is done, as will be seen in the following sections, one can obtain rich information on the process and on the catalyst and its environment.

### 4.2. Micellar Solutions

As mentioned previously, the kinetics of microheterogeneous catalysis when one of the reactants is in an excited state is complicated by the coupling of the reactive and diffusive steps. This complication, however, gives information on the characteristics of the diffusive steps. In order to explore this field, several treatments have been developed.

Thus, Infelta *et al*. [134,135] considered the case in which the reactant, in its excited state, is present only in the micellar pseudophase and the quencher is distributed between the two pseudophases present in the system:

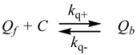
(153)


Under these circumstances, the decay of R^*^ follows the equation:

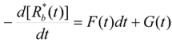
(154)
where *F*(*t*) represents the rate of decay of R^* ^in the absence of the quencher, given by:

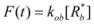
(155)
and *G*(*t*) is the contribution of the reaction (quenching) to the decay of R_b_^*^:

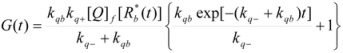
(156)
where [*Q*]*_f_* represents the quencher concentration in the aqueous pseudophase. From the previous equations it follows that:

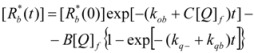
(157a)

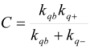
(157b)

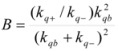
(157c)


The previous equation gives an idea of the complexity resulting from the coupling of the reactive and diffusive events. However, these equations simplify under some circumstances. Thus, for long times, when 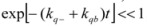, it follows that:

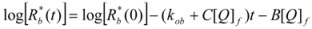
(158)


This equation, working at different [*Q*]*_f_* , permit us to have *C*, *B* and *k_ob_* and, once one has determined these parameters, it is possible to calculated *k_q+_* and *k_q-_*:

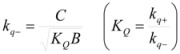
(159a)

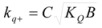
(159b)
(notice that *K_Q_* can be determined in an independent experiment). So, all the rate constants determining the kinetics of the decay of R_b_^*^ can be obtained.

Infelta *et al*. considered other scenarios, but as in the example considered previously, it is always possible, by selecting the conditions of the experiments, to determine all the kinetic parameters in the system. In fact, the model of Infelta *et al.* is an approximate model, as pointed out by Barzykin and Tachiya, due to the fact that a Poisson occupation of the micelles is assumed, and many-body effects, which can be important at high micelle concentration, are neglected. However, Barzykin and Tachiya [136] concluded that the approximate Infelta model works fairly well in most of the cases.

Almgren *et al*. [137] considered the situation in which the reactant can exchange its location whereas the quencher is only soluble in the aqueous pseudophase:

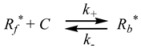
(160a)

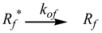
(160b)

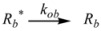
(160c)
and, when the quencher is added to the system:

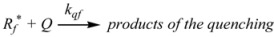
(161)


According to this reaction scheme,

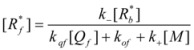
(162)
and the decay of [R_b_^*^(*t*)] is given by:

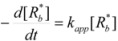
(163a)

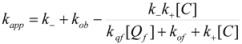
(163b)


This equation permits us to obtain the different rate constants working at different concentrations of *Q* and/or *C*. Extensions of the Almgren model to other scenarios can be found in references [137] and [138]. 

Another reaction scheme was considered by Quina and Toscano:

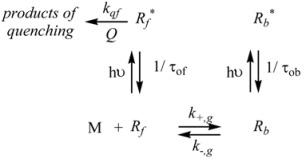
(164)


In this case there is no exchange between R^*^ in the micellar and aqueous pseudophases, that is, the situation corresponds to the slow exchange limit. If, as assumed by Quina and Toscano, the quencher has no access to the bound reactant, it is obvious that the decay of R_b_^*^ is independent of the quencher concentration. Thus, for the aqueous pseudophase the Stern-Volmer equation holds:

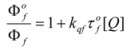
(165)


If the excitation wavelength corresponds to the isosbestic point of the absorption spectrum (in the presence of micelles), the total quantum yield, in the absence of the quencher, is:

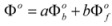
(166)
and when the quencher is present:

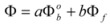
(167)


From the previous equations it follows that:

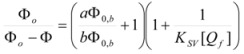
(168)


Quina and Toscano also considered the case in which exchange in the excited state is possible, but this exchange is not rapid enough to be at equilibrium:

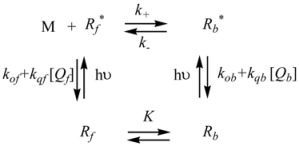
(169)


If, as the authors assumed, [*Q_f_*] = [*Q_b_*] = [*Q*], the apparent rate constant for the fluorescence decay is given by:

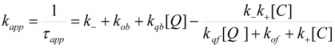
(170)


In this equation *k_of_* and *k_qf_* can be obtained directly from experiments in the absence of receptors, *C*. The other rate constants in Equation 170 can be obtained directly from data corresponding to experiments at different [*C*] and/or [*Q*]. 

The equations of Quina and Toscano have been checked in micellar solutions [139] as well as in bile salt aggregates [140,141] and cyclodextrins [142]. All previous models for processes in micellar solutions have been checked many times with experimental results. According to this, it seems reasonable to conclude that these models work well provided that their basic assumptions are accomplished in the system under study. However, micellar solutions with more than two components can have complex behavior, with complicated phase diagrams. It is clear that, if one moves to different regions of the phase diagram, the behavior of the reaction can change, this reflecting the structural complexity of the system. This circumstance is clearly seen in the case of the anthracene quenching by *N*,*N*-diethylaniline in the SDS/benzyl alcohol/water system [143].

In the opposite extreme of simplicity, one can mention the quenching of the excited state of [Ru (phen)_2_bps] by 4,4’-diheptylviologen in the presence of SDS, studied by Hackett and Turro [144]. This system was selected to obtain “the electron transfer rate constants at the micelles surface without the use of complex equations”. In this case, the neutral ruthenium complex is expected to reside in the aqueous phase, whereas the quenchers are expected to be anchored to the hydrophobic interior of the micelles through their heptil chains, with their redox active groups at the surface of the micelles. So the quenching is only possible at this surface.

In relation to electron transfer processes, other authors studied this kind of reaction in micellar systems, using the Marcus treatment [2].^. ^Thus, Dressik *et al*. [145] have studied the oxidation of Ru(II) photoexcited complexes in micellar solutions. According to them, there is an increase of the reorganization free energy as a consequence of the interaction complex/micelle. This behavior has been observed by other authors [11]. On the other hand, Weidemaier *et al*. [146,147,148] have developed a model for electron transfer at micelles. This model can explain the influence of the length of the surfactant on the reorganization free energy of the electron transfer process.

Barzikyn and Tachiya [149] have also studied the problem of electron transfer reactions in micellar systems. They presented a theory of diffusion assisted reaction on the micelles’ surfaces and applied this treatment to photoinduced electron transfer. The results of the treatment can be obtained in a closed matrix form that, according to the authors, can describe virtually any reaction in any geometry. A relatively similar treatment has been applied by Ranagathan *et al*. [150].

### 4.3. Polymer Solutions

Polymers can act as catalyst showing behavior similar to micelles, as established in section *3.3*. However, the interest in photochemistry and photophysics of ligands bound to polymers comes not so much in relation to catalysis as in relation to some applications such as the fabrication of sensors [151]. Moreover, in many cases photochemical behavior has been studied under conditions in which the ligand is covalently bound to polyelectrolytes. Thus, Morrison *et al*. [152] studied the photochemistry of arenes covalently bound to polyelectrolytes (so, only R_b_^*^ is present in the system), when the quencher is distributed between the polymer and the continuous phase. In this case, the arenes can be placed in different kinds of sites, in the sense that they are not equally accessible to the quencher. These differences in accessibility, in this case, are the consequence of conformational changes of the polymers, in such a way that the photochemistry of the ligand gives structural information on the polymer. It is interesting to note that, in this case, for low quencher concentration, Stern-Volmer behavior is observed. However, when the quencher concentration increases, deviation of this behavior is observed, in such a way that:

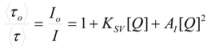
(171)


Correspondingly, the fluorescence decay is monoexponential at low quencher concentrations and multiexponential at higher concentrations.

According to the authors, there are two possible causes for this behavior: (1) conformational changes in the receptor, caused by the quencher, or (2) a non-homogeneous quencher distribution close to the polymer. Calling *A* and *B* the two sites and using a two state model in which the probe is protected from the quencher at site *B*, the authors derived the following equation:

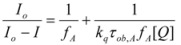
(172a)

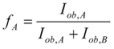
(172b)
which is in agreement with the experimental facts.

On the other hand, Chu and Thomas [153] also studied the quenching of chromophore covalently bound to the polyelectrolyte. The chromophore (pyrene) was located in poly(methacrylic acid) in three different ways: (*a*) as a guest molecule by simple solubilization in its closed compact form; (*b*) covalently bound to the polymers ends and (*c*) randomly bound to the polymer chain (with no more than one pyrene unit per polymer chain in this and *b* cases). In this case, the charge of the polymer and its conformation can be changed through pH variations. As quenchers, Tl^+^ and I^−^ were used. These quenchers behave similarly when the polymer is in compact conformation (until pH = 4). However, once the polymer opens, there are differences between Tl^+^ and I−. In the first case, the negative charge of the polymer increases the local concentration of the quencher, which leads to a dramatic increase of the quenching. On the contrary, iodide ions are repelled by the open (negatively charged) polymer, thus decreasing the quenching process.

The same system, pyrene/poly(methacrylic acid) was also studied by Turro *et al*. [154,155] and at higher pyrene density (on the polymer), in such a way that pyrene/pyrene interactions were possible. As quenchers, nitromethane, Tl^+^, Cu^+2^, I^− ^and 4-dimethylaminopyridine were employed. Additionally, the quenching of pyrene bound to the monomer was studied. In the latter case, plots of *I_o_*/*I vs*. [*Q*] were linear for all the quenchers, but the Stern-Volmer constants only agreed with those from τ_o_/τ *vs*. [*Q*] when *Q* = nitromethane, Tl^+^, and I^−^ but not in the other cases.

The behavior of these two quenchers was attributed by the authors to static quenching in spite of the linear character of their corresponding Stern-Volmer plots. In fact, a case of static quenching would imply:

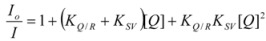
(173)
where *K_Q/R_* is the binding constant fluorophore/quencher (in the ground state) and *K_SV_* the true Stern-Volmer constant (that would be obtained from τ_o_/τ *vs*. [*Q*]). 

Now consider the case of an efficient quencher present at a low concentration. In this case the term containing [*Q*]^2^ can be small, in such a way that a linear Stern-Volmer plot would be obtained. When pyrene is bound to polymer (instead of monomer) the quenching is less efficient, which was considered to be a consequence of the slow diffusion of the quenchers towards R_b_^*^ in the case of the polymer (this implies a total or partial control by diffusion). In this case, differences between the two types of quenchers also appear, the Stern-Volmer plots being linear for nitromethane, Tl^+^ and I^−^ and curved for Cu^+2^ and 4-dimethylaminopyridine. Excimer fluorescence is also observed due to the intrachain interaction of pyrene groups [154,155]. In fact, polymers can produce an enhancement of formation of excimers and exciplexes if they produce an increase of the local chromophore concentration. This question has been considered by Turro *et al*. [156] who studied the influence of poly(styrene sulfonate) on the excimer formation of (α-naphtylmethyl)ammonium chloride. The kinetic analysis of excimer formation, in pure water and in solutions containing the polymer, was made from the following reaction scheme:

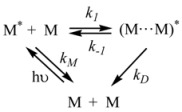
(174)


According to the authors the decay of fluorescence of monomer, *I_M_*, and excimer, *I_D_*, are given by:

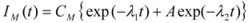
(175a)

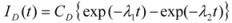
(175b)


*C_M_* and *C_D_* being the concentrations of monomer and excimer, respectively and *A*, *λ_1_* and *λ_2_* are the function of the rate constants shown in the scheme. In fact, *λ_1_* and *λ_2_* can be obtained from the decay curves. From them, taking into account that:

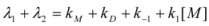
(176)


*k_1_* can be obtained from the (linear) plot of *λ_1_* + *λ_2_* *vs*. [*M*]. Results in this paper reveal that the polymer increases *k_1_* by a factor of 10^3^, although its effect on *k_-1_* is insignificant.An interesting case in which R^*^ is long-lived enough for maintaining the equilibrium distribution between R_b_^*^ and R_f_^*^ has been studied by Wensel *et al*. [157] who considered the effect of DNA on the rates of energy transfer between ions. According to the authors, in the circumstance of rapid exchange between R_b_^*^ and R_f_^*^ the decay is monoexponential, as given by:

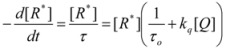
(177)
in such a way that, in the presence of DNA the following equation holds:

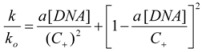
(178)


*k* being the decay rate constant for a given quencher concentration and *k_o_* = 1/τ_o_. *C*_+_ is the total concentration of cations (*R*, *Q* and counterions). On the other hand, Arkin *et al.* [158] report the quenching of photoexcited [Ru(phen)_2_ddpz]^+2^, which binds by intercalation by [Ru(phi)_2_bpy]^+3^ and [Ru(phe)_2_phe]^+3^. The Stern Volmer equation holds for these processes in micellar (SDS) solutions, but the plot of *I_o_*/*Ivs*. [*Q*] are curved in the presence of DNA, as corresponds to a static quenching process. In the case of micelles, the observed rate constant is given by:

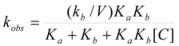
(179)
where* V* is the partial molar volume of the surfactant in the micelle and *K_a_* and *K_b_* are the binding constants of the fluorophore and the quencher to the micelle. The differences observed for DNA and micellar solutions are indicative of the fact that the structure of DNA plays a central role in mediating electron transfer reactions: the π-stack of DNA permits an intimate electron coupling between the donor and the acceptor which is absent in the micelles.

In relation to photoinduced electron transfer between intercalated reactants in DNA, Fukuzumi *et al*. [159] investigated the driving force dependence of the quenching (by electron transfer) of ethidium bromide to and a series of intercalators. They observed that the (electron transfer) quenching rate constants showed behavior in agreement with the Marcus treatment of nonadiabatic intramolecular electron transfer reactions. By using intercalators with different redox potentials they showed that some of these reactions were in the so-called inverted region, that is, *k_q_* decreases as the driving force increases.

Additional complications that the use of polymers as receptors introduces into the system (cooperativity and conformational changes being the most stressed), are reflected in the lower number of papers concerning this topic compared to the number concerning micelles. Besides, the bulk of research is based on the development of polyelectrolyte theories or systems to probe biological important polymers, such as DNA. 

### 4.4. Solutions Containing Saturable Receptors

As mentioned above when dealing with the effects of this kind of receptor in processes in which the reactant(s) are in the ground state, for a given guest molecule, cyclodextrins and related receptors can influence reactivity in different ways. One of the factors influencing the action of this kind of catalyst is the cavity size of the host. This is clearly seen in the work of Yorozu *et al*. [160] who studied the effect of α, β and γ cyclodextrins on the photochemistry of β-naphthol. In this case, given the lifetime of the excited reactant, the excited naphthol decay in the same situation (bound or free) where it appears. Under these circumstances, parameters *a_b_* and *a_f_*, defined in Equation 151b, are the same (*a_b_* = *a_f _*= 1) in such a way that the distribution of free and bound naphthol in the excited state is the same as in the ground state. This is supported by the fact that the distribution of the β-naphthol determined from absorbance measurements is the same as fluorescence techniques.

The authors performed their studies in two limiting situations: (*i*) [*R_f_*] = [*R*], [*R_b_*] = 0 and (*ii*) [*R_b_*] = [*R*], [*R_f_*] = 0 (at a high enough cyclodextrin concentration for encapsulating all the naphthol). The acid dissociation constant of β-naphthol (K_a_^*^) decreases in the presence of cyclodextrins and depends on the type of receptors (α-, β- or γ-CD). However, the diminution of K_a_^*^ is different for the three receptors ((K_a_^*^)_α_<(K_a_^*^)_β_<(K_a_^*^)_γ_), which is due to the differences in the inclusion geometry: for α-CD the OH group of naphthol is placed in the cavity of the receptor, whereas in the case of β- and γ-CD the OH group is partially exposed to water and more so for γ-CD. The authors also studied the quenching of β-naphthol by iodide ion. In this case, the naphthalene group is more exposed in α-CD and the quenching is more effective for this receptor.

The presence of substituents also has influence on the action of cyclodextrins. Thus, Andrade*-*Dias *et al.* [161] have shown the differences between the interactions of sodium decanoate with native and methylated cyclodextrins. According to the authors, the differences arise from the distorted conformations associated with the strong tilt of the axes of one or two glucopyranose units which, in turn, reduces the surfactant access to the cavities and the available space for inclusion.

A complication of the interpretation of photochemical (as well as thermal) data in cyclodextrins solutions arises when there is the possibility of formation of several complexes. Thus, naphthalene and β-CD can form two kinds of inclusion complexes, 1:1 and 2:2. Under these circumstances, the quenching of the excited state of naphthalene by anionic quenchers (I^-^ and IO_3_^-^) was analyzed by Hamai [162] who obtained the quenching rate constants for the different kind of complexes.However, Nelson and Warner [163] considered the quenching of naphthalene in solutions containing β-CD and 1% (v/v) of benzyl alcohol. The data can be interpreted considering that only the 1:1 complex is formed. According to the authors:

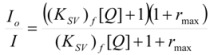
(180)
where *r_max_* is given by:

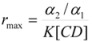
(181)


*α_1_* and *α_2_* being the proportionality constants defined as:

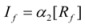
(182a)

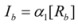
(182b)


These equations hold in the presence of the alcohol, but not when it is component is absent.

An interesting possibility arises when both, the quencher and the fluorophore can be included in the same cyclodextrin, this leading to the acceleration of the (bimolecular) quenching. This possibility was first reported by Kano *et al.* [164] and later by other authors. [165,166,167]. The formation of a ternary complex *R*/*CD*/*Q* is clearly shown by the strong component of the static quencher, not observed in the absence of CD [168]. These kinds of ternary complexes are of interest in relation to analytical applications. Thus, 1-bromonaphthalene has been studied as a probe for detection of menthol [169].

Cyclodextrins, contrary to polymers, are receptors with a well-defined structure in a great range of concentration and environments. Their cavities are suitable to accommodate a great variety of luminescent guests with a well-defined stoichiometry. For these reasons, cyclodextrins are quite adequate receptors to check kinetic models. Besides, the possibility of having substituted cyclodextrins (becoming them in charged receptors, for example) offers a wide framework for research. For these reasons, cyclodextrins are quite adequate receptors to check kinetic models. 

### 4.5. Complex Systems

As established in Section 4a the main interest in the field of microheterogeneous catalysis with participation of excited states has been to developed artificial photosynthetic systems that could reproduce behavior of the natural ones. In photosynthetic process one of the key steps is a photoinduced charge separation:

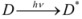
(183a)

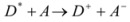
(183b)


Processes *a* and *b* are rapid reactions. Thus, the main problem is not to produce a charge separated state. However, in most of the cases, reaction (183b) is followed by the (also rapid) charge recombination (the back reaction):

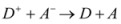
(184)


**Scheme 2:**
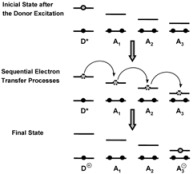
Simplified scheme of sequential electron transfer process in a photosynthetic system.

This reaction implies that the energy accumulated in the *D*^+^ + *A*^−^ pair is lost. In this regard, a good part of the efforts for producing artificial photosynthetic systems has been related to the production of donors (*D*) or acceptors (*A*) that produce long-lived charge separated states. This part of the process has reached important advances. Thus, Fukuzumi *et al*. [170] have prepared donors and acceptors that participate in photoinduced electron transfer processes leading to products with lifetimes longer than the lifetime of natural systems, and accumulating a higher energy. These two characteristics are important but something more is necessary: although the *D*^+^ + *A*^−^ pair was long-lived, it is necessary to extract the energy accumulated by this pair before the back reaction happens. A way to obtain this stabilization is to maintain a series of acceptors (or donors) close to the primary pair able to experience a charge transfer process. This situation is depicted in the Scheme 2.

It is clear from this scheme that in the successive electron transfer there is a loss of energy. But, at the same time, the distance between the constituent, *D*^+^ and *A*^−^ pair, increases. Given that the rate of an electron transfer decreases exponentially with this distance [171] the energetic loss is compensated by a much longer lifetime, long enough to permit the energy transfer in the correct direction and, thus, opening the possibility of been stored. An important question is that, in order to reach these objectives, the donor and the acceptors must be fixed to avoid their diffusional movements that could produce the contacts *D*^+^/*A*^-^, with the subsequent back electron transfer. For this reason, in the natural systems, *D* and *A* are fixed in the cellular membranes. In artificial photosynthetic systems, consequently the *D*/*A* system must be fixed to some kind of membrane. As an example the system developed by Gust *et al*. [125] will be considered. This system is constituted by a triade having a quinone, a porphyrin and a carothene (1). It is placed in an artificial membrane (a vesicle) together with a second quinone (2).

**Scheme 3:**
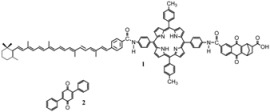
Formulas of the triade and the quinone employed in reference 125.

The triad (1) is inserted into the membrane vectorially, with the quinone pointing towards the exterior of the vesicle. After excitation of the porphyrin, there is a charge separation process, in such a way that the quinone acquires a positive charge whereas the quinone in the triad acquires a negative charge. After this, the free quinone (2) is reduced by the quinone in the triad, producing a semiquinone anion. This anion can accept a proton from the solution where the vesicles are dispersed. The protonated (and neutral) quinone can move freely in the membrane. If this quinone contacts the carothene, it is oxidized forming a protonated quinone positively charged, which is acidic, pKa = −6. This protonated quinone can release a proton into the interior of the vesicle, in such a way that a protonic gradient appears. The free energy corresponding to this gradient, that is, to the difference of proton chemical potential, is used to produce ATP from ADP and phosphate. The yield of the system is relatively low, one ATP molecule is produced for every fourteen incident photons. However, it is a promising system that opens the possibility of future improvement.

There are alternatives to the production of fuels in artificial photosynthetic systems. Some of these alternatives have been considered in a recent review article by Gust *et al*. [172]. The alternatives go from systems able to produce ATP employing systems like those previously described to other systems devoted to photoreforming of biofuels to hydrogen. The latter systems employ photochemical biofuel cells. These cells consume biofuels such as glucose, ethanol or methanol to generate hydrogen. The photoactive system is the anode of the cell constituted by a glass covered with a transparent conductor where a thin layer of nanoparticles of SnO_2_ or TiO_2_ are inserted. These nanoparticles, in turn, are covered with a sensitizer (a porphyrin). The resulting electrode is immersed in a solution containing NADH/NAD^+^ (NADH = β-nicotinamida adenine dinucleotide). Upon illumination, the excited porphyrin, P^*^, inject an electron into the electrode, generating *P*^+·^. These electrons flow to the cathode through an external wire: in the cathodic compartment (which is separated from the rest of the solution by an ion-selective membrane that allows hydrogen ions to diffuse through the solution) protons are reduced to hydrogen. On the other hand, *P*^+· ^are reduced in the anodic compartment by NADH to produce *P* and NAD^+^. In the anodic compartment, NAD^+^ is reduced by a dehydrogenase enzymatic catalyst, in such a way that the oxidized form of the enzyme oxidizes the biofuel. In this way, the cell functions to reform the biofuel, and hydrogen is produced through a reduction of H^+^, employing electrons that, at the end, come from the biofuel.

An interesting photochemical device is based on the idea of employing water instead of biofuel. In this case, the biofuel oxidation catalyst is replaced by a water oxidation catalyst (nanoparticle IrO_2_·nH_2_O). Also the porphyrin is replaced by ruthenium polypyridyl dye, which is colled to the electrode through adequate functional groups. 

The principle of operation in this cell is similar to that of the photochemical biofuel cell. In this case, the electrons injected into the anode by the excited ruthenium complex again are employed (in the cathode) to reduce protons to hydrogen. On the other hand, the oxidized ruthenium complex is reduced by the iridium oxide nanoparticles, which, in turn, oxidize water to O_2_ and protons. This solar water splitting system is clearly inefficient and unstable to be useful for practical purposes. Nevertheless, the system has the possibility of hydrogen production via water oxidation, using ideas drawn from natural photosynthesis and molecular photochemistry. 

Other strategies for solar energy conversion have been developed. Among these is the approach based on dye sensitized solar cells that involve the immobilization of a single molecular specie to nanocristalyne TiO_2_. Choi *et al.* [173] have demonstrated that the yield of these cells can be increased if the dyes are, in turn, encapsulated by cyclodextrins. An interesting consequence of the encapsulation is an increase of the stability of the system.

As we have seen nanoparticles are crucial components of the systems described previously for solar energy conversion. Other applications of nanoparticles in the field of catalysis are also possible: thus, in the field of chemical catalysis they offer interesting possibilities since chemical selectivity can be determined controlling the shape and size of the nanoparticles. An excellent review on this topic can be found in reference [174].

A conclusion emerges from the previous paragraph. Although simple systems constituted by only one kind of receptors can be useful, and they are indeed useful to discover the basic characteristics of microcatalysis, it appears that from a practical point of view, more complex systems will be needed probably to achieve some functions, such as photochemical water splitting. This would actually open new doors to basic and applied research. In this regard, the rapid development of new experimental techniques will play an important role [174].

## 5. Conclusions

In this work the topic of microheterogeneous catalysis has been reviewed.The interest in microheterogeneous catalysis arises from different reasons. On the one hand, it can mimic catalysis of biological systems. On the other hand, microheterogeneous catalysis is also of interest in the fields of inorganic and organic synthesis in order to obtain a desired product, favoured in the presence of a microheterogeneous system. The main research of the microheterogeneously catalyzed reactions in the excited state has been centred on finding practical systems for solar energy conversion and storage. 

It has been shown that the equations describing microheterogeneous catalysis are particular cases of the Brönsted equation and can be derived from the latter if one expresses the activity coefficient taking the correct reference state. Besides, catalysis in the homogeneous media, as well as in the heterogeneous media and the fundamental equation in the field of the electrocatalysis can also be derived from the Brönsted equation. 

When in the catalyzed reaction the reactants are in their ground state, the Pseudophase Model has been used extensively because this formulation is flexible enough to take into account the peculiarities of each system. For example, in micellar solutions, the effects on the rate constants of the ion condensation at the micellar surface can be taken into account through decomposition of the binding constant in an electrostatic and a non-electrostatic component. This separation can be accomplished by using Lippard’s equation. The effects of the ion condensation have also been focused from other points of view. In this regard, Quina and Chaimovich developed the Ion Exchange Pseudophase Model, and Rodenas *et al*. studied this effect employing a treatment based on a cell model and the non-linearized Poisson-Boltzmann equation. The effect of ion-pair formation between the reactants can be also taken into account through a formal modification of the Pseudophase Model formulation. 

At premicellar concentration, a sigmoidal dependence of the rate constant on the substrate concentration is observed. This behavior, also observed in enzyme solutions, has been rationalized by Piszkiewicz and Biresaw-Bunton. 

In reverse micelles, a modified Pseudophase Model which takes into account the number of binding sites in a droplet has been employed satisfactorily.

For saturable receptors, the Pseudophase Model can be modified to take into account the existence of four possible reactions paths when both of the reactants can bind to the receptors. 

Cooperative effects, present in polymer solutions, can be introduced in the Pseudophase Model expressing the binding constant as a parameter which depends on polymer concentration.

Use of the Pseudophase Model requires that the free and bound reactants are in equilibrium. However, in photochemical processes this premise is not always accomplished. It depends on the relation between the lifetime of the excited state and the rates of the forward and reverse processes in the union to the catalyst. However, within the limits of fast or slow exchange, the Pseudophase Model holds, at least formally. For other situations, the most important models treating the main possible scenarios have been reviewed. Basically, the Infelta model treats the case in which the excited species are present only in the micellar pseudophase and the quenchers are distributed between the aqueous and micellar pseudophases, following a Poisson distribution. In the Almgren model there are two cases. In both, the excited species are present in aqueous and micellar pseudophases but the quencher is only present in the aqueous pseudophase in one case and, in the other, the quencher is also present in the micellar pseudophase. The differences between these models arise from the quencher distribution in a given micelle containing one excited probe. On the other hand, the Quina model treats the cases in which the exchange of the probe is or not possible. These models were developed to study photochemical reactions in micelles as receptor, but their applicability to polymers and cyclodextrins has also been considered. 
